# Distribution analysis of the finless porpoises (*Neophocaena* sp.) and oceanic dolphins (Delphinidae) in the Korean Sea using environmental DNA

**DOI:** 10.1371/journal.pone.0322148

**Published:** 2025-05-16

**Authors:** Sung Bin Lee, Byung Yeop Kim, Won Joon Jung, Han Seok Cho, Kevin Cho, Hyemin Kim, Euna Song, Sib Sankar Giri, Su Jin Jo, Mae Hyun Hwang, Jae Hong Park, Da Sol Park, Eun Jae Park, Ihn-Sil Kwak, Se Chang Park

**Affiliations:** 1 Laboratory of Aquatic Biomedicine, College of Veterinary Medicine and Research Institute for Veterinary Science, Seoul National University, Seoul, Korea; 2 Department of Marine Industry and Maritime Police, College of Ocean Science, Jeju National University, Jeju, Korea; 3 College of Veterinary Medicine, Seoul National University, Seoul, Korea; 4 Department of Agricultural, Consumer, and Environmental Sciences, University of Illinois at Urbana Champaign, Champaign, Illinois, United States of America; 5 Department of Ocean Integrated Science, Chonnam National University, Yeosu, Korea; Charles University, CZECHIA

## Abstract

Environmental DNA (eDNA) serves as a non-invasive tool for monitoring the presence of specific organisms in challenging or hard-to-access areas. We attempted non-invasive monitoring of Korean cetacean species by extracting eDNA from the western and southern seas of the Republic of Korea, as well as around Jeju Island. In the present study, we focused on two representative cetaceans of the Korean Sea: the narrow-ridged finless porpoise (*Neophocaena asiaeorientalis sunameri*) and oceanic dolphins (Family Delphinidae). When selecting polymerase chain reaction primers, mitochondrial DNA (mtDNA) of *N. asiaeorientalis* and microsatellite Slo4 of oceanic dolphins were identified as the most effective gene sequences in high abundance in low concentration eDNA samples, using tissue samples for eDNA detection of the target species. A total of 139 samples were collected, and eDNA was detected from finless porpoises (*Neophocaena* sp.) in 94 samples (68%) and from oceanic dolphins in 50 samples (36%). Significantly, eDNA revealed the considerable presence of finless porpoise around Jeju Island, despite a lack of visual confirmation. In the Yellow Sea, eDNA primarily detected the presence of common dolphin (*Delphinus delphis*), orca (*Orcinus orca*), and Indo-Pacific bottlenose dolphin (*Tursiops aduncus*). Indo-Pacific bottlenose dolphins were identified along the coast of Jeju Island. The value of this research lies in being the first attempt to explore cetacean eDNA across various species in Korea. Further cetacean eDNA research should focus on developing metabarcoding primers capable of detecting a greater variety of cetacean species and primers for detecting specific porpoise species. This study will serve as a valuable reference for future studies.

## Introduction

Cetaceans play a pivotal role in maintaining species diversity within marine ecosystems as keystone species and serve as sentinel animals that indicate the severity of environmental pollution [1–4]. Among these cetacean species, the narrow-ridged finless porpoise (*Neophocaena asiaeorientalis sunameri*, NRFP) is the dominant species inhabiting Korean western and southern coastal waters, along with other species, such as oceanic dolphins (Family Delphinidae) and large baleen whales [5,6]. However, the NRFP population is currently declining rapidly because of bycatch, habitat reduction, and water pollution, leading to its listing as an endangered species on the Red List of the International Union for Conservation of Nature (IUCN) since 2017 [7–9]. Understanding the distribution and population dynamics of NRFP in Korean waters is a major challenge for establishing protected areas and implementing policies for species conservation. Annual cetacean-watching surveys were conducted in the Republic of Korea to estimate cetacean populations [6,10]. According to the survey results, NRFP is primarily distributed in the western and southern seas of Korea, and its distribution is unclear in the waters near Jeju Island, except for the Chuja Islands north of Jeju Island. In contrast, one of the oceanic dolphins, the Indo-Pacific bottlenose dolphin (*Tursiops aduncus*, IPBD), is characterized by its residency and distribution along the coast of Jeju Island [6,10].

Environmental DNA (eDNA) refers to the trace amounts of biological DNA in the environment [11–13]. eDNA is released into the environment through the skin, excrement, and other biological processes in animals. Even in minimal quantities, it can sensitively detect the presence of a species [12,14,15]. It can be utilized in non-invasive monitoring studies to investigate the presence of specific organisms in challenging and hard-to-reach areas [16–18]. Sampling techniques such as kick sampling and surber are invasive methods that can destroy the habitats of aquatic organisms, while non-invasive environmental monitoring approaches like eDNA techniques, which have minimal direct human impact, have recently gained attention in the academic community [12,19,20]. In the Republic of Korea, eDNA research is actively conducted in fields, such as biological monitoring within the demilitarized zone (DMZ), which is difficult for people to access; detection of DNA from endangered fish species in freshwater and seawater; and locating spawning areas for fish [21,22]. While various studies on cetacean eDNA detection are being conducted internationally [23–26], there has been limited exploration in the realm of cetacean eDNA in Korean seawaters, with very few attempts thus far. In addition, cetacean-watching surveys are costly, time-consuming, and subject to temporal and spatial constraints that limit their effectiveness [27]. Therefore, using eDNA for distribution analysis is a viable method to mitigate these economic, temporal, and spatial limitations [28].

In this study, we used eDNA to examine whether the DNA of NRFP was detectable in the waters surrounding Jeju Island, the West Sea, and the South Sea and confirmed the presence of various oceanic dolphins (family Delphinidae), including IPBD. The microsatellite Slo4 gene sequences of Jeju IPBD and common dolphin were analyzed in this study and registered in the NCBI GenBank. This study is significant because it is the first to examine the eDNA of small-toothed cetaceans in Korean waters, allowing us to create a distribution map and gain insights into their approximate distribution.

## Materials and methods

### Field surveys with seawater sample collection and filtration

Field surveys and seawater sampling in the western and southern Korean seas were conducted aboard the Ara-ho, the research vessel of Jeju National University [29] and coastal samplings of the Jeju Island were conducted on foot. Sampling was primarily conducted during the summer season, as previous research has indicated higher detection of eDNA during summer than winter [30,31] and following vessel operational schedule. Sampling activities were conducted a total of seven times, and they were categorized into Areas A to G based on the sampling dates and locations. Field surveys and sampling activities of the Jeju coastline were conducted on foot at 39 coastline points in July 2022 (Area A) and at 19 points in July 2023 around Jeju Island (Area B). These locations were selected based on the safety of the researchers and their accessibility to humans. In addition, sites with minimal human traffic were chosen to maintain an approximate distance of 4 km between sites. Intensive sample collection efforts were focused on Daejeong-eup and Gujwa-eup, where IPBD (*Tursiops aduncus*) are frequently found on Jeju Island [32,33]. In June 2022, 13 samples were collected from the waters near Chuja Islands (Area C) and in June 2023, 24 samples were collected from Southern Sea and Jeju Sea (Area D) with the vessel. In May 2022, 18 samples were collected from the Yellow Sea (Area E), and in May 2023, 20 samples were collected from the Yellow Sea (Area F) with the vessel. In June 2023, additional six samples were collected from the southern sea of Jeju Island (Area G) with the vessel. Sampling in 2022 and 2023 was conducted with the aim of repeating at the same locations as much as possible. However, in Areas C, D, and G, the sampling locations were different due to the vessel operating routes. No permits required for access, since all sampling was conducted within Korean waters.

Seawater samples were collected in new 1-L plastic bottles from the surface (< 0.5 m) upon arrival at each sampling point. At each point, 100 mL of surface water was collected ten times to accumulate a total of 1 L of water. The nitrile gloves were replaced after each sampling event. To eliminate bacteria and germs from the surfaces of the sample containers and to prevent cross-contamination between samples, a 10% bleach solution was applied with bathing, followed by rinsing with distilled water and air-drying. The water samples were promptly subjected to filtration using Durapore® 47 mm Hydrophilic PVDF Membrane Filter with a pore size of 0.45 µm (Merck Millipore, MA, USA), KNF Laboport® solid PTFE vacuum pump (KNF Neuberger, NJ, USA), and solid suspension filtering apparatus CUOA0176 (CuotaLab Sharing Korea, Seoul, Republic of Korea). The intervals between successive filtering processes were addressed by rinsing the apparatus with 10% bleach solution, tap water, and distilled water. Following the filtration procedure, the membranes were preserved in 100% EtOH with sodium acetate [34,35].

### DNA extraction of eDNA samples and positive controls

The filter membranes were dissected into 1-mm fragments and immersed in a 1 × PBS solution containing 10% proteinase K. Bead-beating extraction was performed for 20 min using the OMNI Bead Ruptor 12 and 1.4 mm Ceramic Tube (OMNI International, GA, USA) [36]. Subsequently, the samples were subjected to incubation at 56.5 °C using a heat block, and the eDNA was extracted using the GeneJET Genomic DNA Purification Kit (Thermo Scientific, MA, USA).

Skin and spleen biopsy samples collected from NRFP (*N. asiaeorientalis*), Indo-Pacific finless porpoise (*N. phocaenoides*), and oceanic dolphins such as IPBD (*T. aduncus*) and common dolphin (*Delphinus delphis*) were used as positive controls for eDNA analysis. These cetacean species were stranded along the coast of Jeju Island during March to April 2022, and the necropsy and sampling of these carcasses were conducted by veterinarians of Seoul National University from July 17–20, 2022, at the Jeju office of the Korean Fisheries Resources Agency (FIRA; 23 Ongpo 7-gil, Hallim-eup, Jeju, Republic of Korea). The skin samples were dissected into 1-mm fragments, and all other procedures were carried out identically to the eDNA sample processing protocol described above.

### Primer design and selection

Primer design was based on the mitochondrial DNA (mtDNA) cytochrome b gene sequence of the NRFP (KU820912) and IPBD (AF084092) which are the main target species in this study and available in NCBI GenBank, following the methodology [37]. Three primer pairs for the NRFP (comprising forward and reverse primers of EAFP1, 2, and 3) and five primer pairs for the IPBD (including forward and reverse primers of IPBD1, 2, 3, 4, and 5) were designed using Primer–BLAST with default settings. The newly designed primers were used to compare and analyze species identification ability and eDNA amplification efficiency alongside primers reported in previous studies to find suitable primers for eDNA experiments. Since research on eDNA of cetaceans in Korean waters has not been attempted before, the development of new primers with amplification efficiency of eDNA was pursued.

Positive controls, negative controls, and previously established primer pairs were tested to assess the efficacy of amplification and species discrimination, including M13-Dlp1.5-L & Dlp5-H [38,39], H5730 & L5652 [40], L15775 & H00651 [41,42], Dlp1.5 & Dlp8 [43], YFP-F & R [30], Slo4-F & R [44,45], and Ceto2-F & R [46].

To evaluate whether it was possible to amplify the eDNA of cetaceans present in extremely low amounts in the samples, the DNA of positive controls was diluted and the efficacy of amplification was tested. Positive controls were diluted in 1 × PBS solution at dilutions of 10^−1^, 10^−2^, 10^−3^, 10^−4^, and 10^−5^, and polymerase chain reaction (PCR) was performed with each primer pair to evaluate amplification efficacy and species discrimination.

### Conventional PCR examination of eDNA

Two primer pairs were selected based on the results of species discrimination and eDNA amplification. The primer pair, YFP-F (5′-TATGTCCACTAGCCCTTCATAACCATTA-3′) and YFP-R (5′- AGATCATTATTTAGCTACCCCCACAAGC-3′), was designed to amplify the 102-base pair (bp) fragment of *Neophocaena asiaeorientalis* mitochondrial D-loop sequence in the previous study [30]. Furthermore, the primer pair, Slo4-F (5′-CATATTACAACGGTCTTGTAAACC-3′) and Slo4-R (5′-GTCATAAGTCCATCGAGATGTC-3′), was designed to amplify the 212–226-bp fragment of microsatellite Slo4 of the oceanic dolphins (the family Delphinidae) in the previous studies [44,45]. The Slo4-F & R primer pair has amplified microsatellite gene partial sequence from oceanic dolphins such as the IPBD (*T. aduncus*), spinner dolphin (*Stenella longirostris*), Clymene dolphin (*Stenella clymene*), and false killer whale (*Pseudorca crassidens*) in previous studies, confirming its ability to distinguish at the species level.

All samples were examined triplicates using the primer pairs YFP-F and YFP-R to amplify eDNA of *Neophocaena* sp. Around 20 µ L PCR mixture included 1 µ L of sample, 1 µ L of each primers YFP-F and YFP-R, 1 µ L of Maxime™ PCR PreMix (LiliF Diagnostics, Seongnam, Korea), and 15 µ L of nuclease-free water. Conventional PCR test was carried out with the following protocol: initial denaturation at 95 °C for 3 min, followed by 45 cycles, each consisting of a denaturation step at 95 °C for 10 s, an annealing step at 60 °C for 20 s, and an elongation step at 72 °C for 30 s [30]. The final elongation step was performed at 72 °C for 10 min. Both positive and negative controls were used in this study.

All samples were examined triplicates using the primer pair Slo4-F and Slo4-R to amplify the eDNA of oceanic dolphins (Delphinidae family). The main target Delphinidae species of this study were IPBD, orca, and common dolphin. The 20 µ L PCR mixture included 1 µ L of sample, 1 µ L of each primers Slo4-F and Slo4-R, 1 µ L of Maxime™ PCR PreMix, and 15 µ L of nuclease-free water. Conventional PCR test was carried out with the following protocol: initial denaturation at 95 °C for 5 min, followed by 45 cycles, each consisting of a denaturation step at 94 °C for 30 s, an annealing step at 55 °C for 20 s, and an elongation step at 72 °C for 20 s [44]. The final elongation step was performed at 72 °C for 10 min. Both positive and negative controls were used in this study.

### Sequencing and annotation

Each amplified PCR product was resolved using 1% gel electrophoresis with 0.5 µg/mL ethidium bromide to separate the target DNA molecules. DNA fragment bands were visualized using UV transillumination. DNA fragments were extracted using a QIAquick Gel Extraction Kit (Qiagen) and sequenced for further genetic analysis at Bionics Korea (Seongdong-gu, Seoul, Republic of Korea).

The sequences were annotated using standard nucleotide BLAST of the National Institute of Health (NIH) with standard databases; nucleotide collection (nr/nt) and optimization for highly similar sequences (megablast) [47]. Sequences were identified by assignment to a database sequence with query cover (%) and percent identity (%).

### Establishment of standard curves

DNA extracted from skin and spleen biopsies of NRFP and IPBD was used as positive control. The concentration of the extracted DNA was measured and serially diluted to achieve concentrations of 5 × 10^4^, 5 × 10^5^, 5 × 10^6^, 5 × 10^7^, and 5 × 10^8^ copies/ µ L. Triplicates of the diluted DNA were used to perform qPCRs with YFP-F & R for the NRFP mtDNA D-loop gene partial sequence and Slo4-F & R for the oceanic dolphins microsatellite Slo4 gene sequence to establish standard curves. Negative controls were used in this study.

### Real-time PCR

The primer pairs YFP-F & R and Slo4-F & R were used to determine eDNA concentration in each sample which has been shown in previous studies to be positively correlated with bio-density, biomass, and timing of occurrence [28,48,49]. Quantitative real-time PCR was performed triplicates using the Step One Plus Real-Time PCR System (Thermo Fisher Scientific, Waltham, MA, USA). PCR was performed in a total 20 µ L volume containing 2 µ L of extracted DNA, 0.8 µ L of each primer (10 µ M), 10 µl of PowerUp SYBR Green Master Mix (Thermo Fisher Scientific, MA, USA), and 6.4 µ L of ddH_2_O. PCR was carried out as follows: 3 min at 95 °C, 45 cycles of 10 s at 95 °C and 20 s at 60 °C, followed by a final melt-curve stage. One positive and one negative control were included in each run, and all qPCR reactions were performed in triplicate. Both positive and negative controls were used in this study.

### Distribution analysis of cetacean eDNA in Korean Seas

The map was illustrated using Adobe Photoshop version 2021 (Adobe, San Jose, CA, USA). During the sampling process, the latitude and longitude of each point were recorded using the Global Positioning System (GPS), and this location data was manually marked on the illustrated map. The geo-information distributed online platforms (GIDOP) Earth and Open Sea Map were utilized to add the direction of ocean currents and water depth [50,51].

### Phylogenetic analysis

Phylogenetic analysis of the *Neophocaena* sp. mtDNA D-loop gene partial sequences was performed in VICTOR [52] with the recommended settings, using three sequences of NRFPs from Jeju Island, two sequences of Indo-Pacific finless porpoises from Jeju Island, 67 D-loop gene partial sequences of finless porpoises from China (GenBank accession numbers AF289348, AF289351, AF481866-AF481873, AY334099-AY334101, HM063475-HM106478, HM106484-HM106486, HQ108428-HQ108437, KC135874-KC135880, KC408440-KC408444, KR108307, KR108308, KT852939, KU820905-KU820907, KU886000, KX650869, KX650871, KX650872, MG719601, MT948058, NC021461, NC026456, OP566539-OP566543, OP566551, OP566561, OP566568, and OP566572-OP566575), 27 sequences of finless porpoises from Korean peninsula (ON470395-ON470438), 2 sequences of Indo-Pacific finless porpoises from Japan (AB610334 and AB610334), and a sequence of Indo-Pacific finless porpoise from Taiwan (MT948060).

Phylogenetic analysis of the Delphinidae microsatellite Slo4 gene sequences was performed in VICTOR with the recommended settings, using Slo4 sequences of IPBD (GenBank accession number OR778395), common dolphin (PQ869633), and spinner dolphin (OR778395, PQ869633, and AM490785) and partial sequences of striped dolphin chromosome 13 (OX596398), orca chromosome 13 (OW443373), and Risso’s dolphin chromosome 15 (OZ206328).

The sequences were aligned with Clustal W method using default settings, then phylogenetic trees were visualized using Maximum Likelihood with 1,000 bootstraps in MEGA X version 10.0.5 [53]. The phylogenetic tree of *Neophocaena* sp. eDNA was visualized with branch lengths included. The lengths of the branches represent the evolutionary distances between the clusters. On the other hand, the phylogenetic tree of Delphinidae eDNA was visualized with topology only, without branch lengths, to better illustrate the relationships between clusters.

### Haplotype network analysis

Haplotype networks of the *Neophocaena* sp. mtDNA D-loop gene partial sequences and *Delphinidae* sp. microsatellite Slo4 sequences were constructed using DNASP version 6 [54], respectively. Genetic diversity and structural patterns of each eDNA individual and population were visualized using PopART Studio version 1.7 based on a Templeton-Crandall-Sing (TCS) algorithm [55].

## Results

### Primer design and selection

Three primer pairs were designed for the NRFP (forward and reverse primers of EAFP1, 2, and 3), and five primer pairs were designed for the IPBD (forward and reverse primers of IPBD1, 2, 3, 4, and 5). Most of the designed primer pairs showed species discrimination, except for IPBD3; however, all the designed primer pairs failed to amplify the DNA of the positive control at low dilution concentrations, such as 10^-4^ and 10^-5^, indicating low efficacy of amplification (Table 1).

**Table 1:** List of primer pairs and the results of species discrimination and efficacy of eDNA amplification tests.

Primer name	Primer sequence (5′-3′)	Melting temperature (Tm; ℃)	Species discrimination; Confirmation of amplified bands and sequencing	Efficacy of eDNA amplification (The lowest dilution concentration at which amplified bands were confirmed.)	Reference
EAFP1-F	AATCCCCAACAAACTGGGGG	60	+^a^	++^b^ (10^-2^)	
EAFP1-R	AGTTGGCTAAAGGGCCGAAA				
EAFP2-F	CCCACAGGAATTCCGTCCAA	60	+	++ (10^-2^)	
EAFP2-R	AGGGTCACCTAGAAGGTCGG				
EAFP3-F	CACACCAGACACCTCTACCG	55–60	+	+ (10^-1^)	
EAFP3-R	AATACAGGCCACGTCCGATG				
IPBD1-F	AATTGGAGGCCAACCCGTAG	60	+	++ (10^-2^)	
IPBD1-R	AGGCCGGCTGTTGGTATTAG				
IPBD2-F	ACGGAGCCTCCATGTTCTTC	59.75	+	++ (10^-2^)	
IPBD2-R	GGCAGGACGTAGCCTACAAA				
IPBD3-F	CCCTATTCACCCCCGACCTA	60	-^c^	–	
IPBD3-R	CTCTGGTTTGATGTGTGCGG				
IPBD4-F	CCCACAGGAATCCCATCCAA	60	+	+ (10^-1^)	
IPBD4-R	CCTAGTAGGTCGGGGGTGAA				
IPBD5-F	GAACCCTAATCGCGGACCTC	60	+	++ (10^-2^)	
IPBD5-R	AATAAGGCCGGCTGTTGGTA				
M13-Dlp1.5-L	TGTAAAACGGCCAGTTCACCCAAAGCTGRARTTCTA	54	+	++ (10^-2^)	[38,39]
Dlp5-H	CCATCGWGATGTCTTATTTAAGRGGAA				
H5730	GCAGGYACAGGYTGAACYG	55	+	++ (10^-2^)	[40]
L5652	GTMTAAAYAAYATRAGCTTCTG				
L15775	GTAAAACGACGGCCATACATGAATTGGAGGACAACCAGT	44	–	–	[41,42]
H00651	TAACTGCAGAAGGCTAGGACCAAACCT				
Dlp1.5	TCACCCAAAGCTGRARTTCTA	53	–	–	[43]
Dlp8	CCATCGWGATGTCTTATTTAAGRGGAA				
YFP-F	TATGTCCACTAGCCCTTCATAACCATTA	60	+	+++++ (10^-5^)	[30]
YFP-R	AGATCATTATTTAGCTACCCCCACAAGC				
Slo4-F	CATATTACAACGGTCTTGTAAACC	50	+	+++++ (10^-5^)	[45]
Slo4-R	GTCATAAGTCCATCGAGATGTC				
Ceto2-F	CACGCACACACCGCCCG	60	+	++ (10^-2^)	[46]
Ceto2-R	GTATGCTTACCTTGTTACGAC				

The designed primers (forward and reverse primers of EAFP1, 2, 3, IPBD1, 2, 3, 4, and 5) were all created using Primer BLAST.

^a^The symbol represents positive results.

^b^The amount of +* *represents the number of amplification reactions that occurred in each sample diluted in five steps from 10^-1^ to 10^-5^ concentrations (minimum of 1, maximum of 5).

^c^The symbol represents negative results.

Previously established primer pairs were also tested. The primer pairs YFP-F & R for the mtDNA D-loop gene partial sequence of NRFP and Slo4-F & R for the microsatellite Slo4 gene sequence of oceanic dolphins showed species discrimination and high efficacy of amplification with 10^−1^, 10^−2^, 10^−3^, 10^−4^, and 10^−5^ positive control dilutions (Table 1). Two primer pairs were selected for eDNA examinations in this study.

### Standard curves

**Fig 1:**
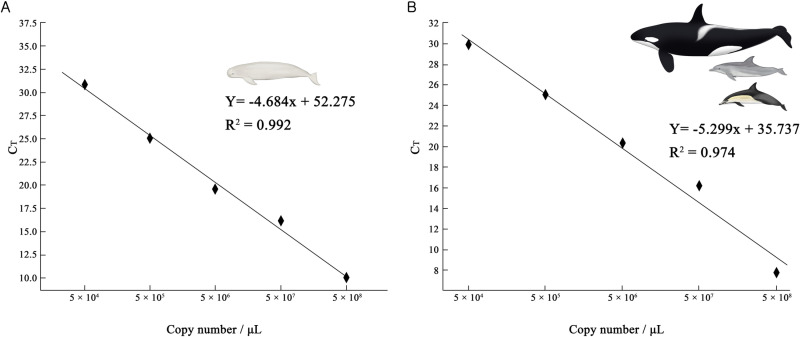
Standard curves of the cetacean gene sequences for examination of eDNA concentration. The x-axis represents the initial amount of DNA before amplification at each dilution of positive controls, and the y-axis represents the threshold cycle (Ct). The slope of the curve and the correlation coefficient (R²) are indicated for each graph. **A** Standard curve of the finless porpoises (*Neophocaena* sp.) mtDNA D-loop gene partial sequence. **B** Standard curve of IPBD microsatellite Slo4 gene sequence. The curve was used for checking the eDNA concentration of oceanic dolphins (Delphinidae) in the samples.

A standard curve of the finless porpoise (*Neophocaena* sp.) mtDNA D-loop gene partial sequence was constructed (y = −4.684x + 39.30, R^2^ = 0.992; Fig 1A) from positive controls extracted from the skin and spleen tissues of the NRFP. Negative controls were used to confirm the lack of amplification.

A standard curve of oceanic dolphin microsatellite Slo4 gene sequence was constructed (y = −5.299x + 35.737, R^2^ = 0.974; Fig 1B) from positive controls extracted from the skin and spleen tissues of IPBD. Negative controls were used to confirm the lack of amplification.

### Conventional and real-time PCR examination of finless porpoise (*Neophocaena* sp.) mtDNA sequence

**Fig 2:**
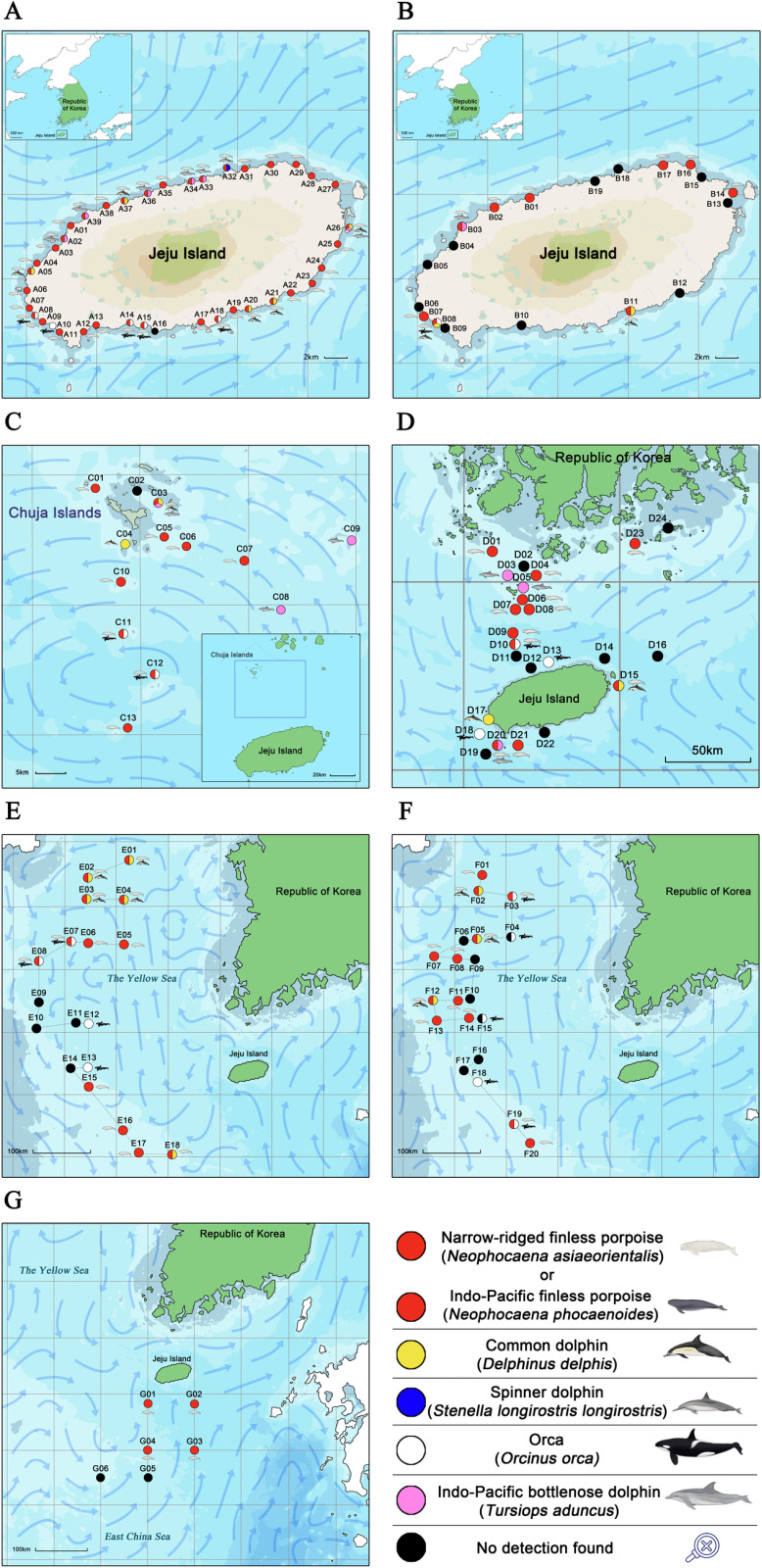
Map of cetacean eDNA examinations using conventional PCR in Korean Seas. The illustration was created by Sung Bin Lee (S.B.L.). **A** Results of samples collected from the coast of Jeju Island in July 2022. **B** Results of samples collected from the coast of Jeju Island in July 2023. **C** Results of samples collected from the Chuja Islands area of Jeju Island in June 2022. **D** Results of samples collected from the southern coast and Chuja Islands area of Jeju Island in June 2023. **E** Results of samples collected from the Yellow Sea in May 2022. **F** Results of samples collected from the Yellow Sea in May 2023. **G** Results of samples collected from the southern waters of Jeju Island in June 2023.

The results of conventional PCR for detecting finless porpoise mtDNA in all eDNA samples are summarized in Table 2, S1 Table, and Fig 2. Of the 139 eDNA samples, mtDNA sequences of the finless porpoise were detected in 94 samples (68%; Table 2). Sequencing results showed that in all positive samples, sequences of the NRFP (*Neophocaena asiaeorientalis*) or Indo-Pacific finless porpoise (*Neophocaena phocaenoides*) were detected. Most positive samples had high values, showing a query coverage of over 99% and identity of over 99% (S1 Table). In all positive samples, the eDNA concentration of the finless porpoise ranged from 10^3^ to 10^4^ copies/ µ L, with particularly high concentrations of 7.37 × 10^4^ copies/ µ L at point A03 and 6.81 × 10^4^ copies/ µ L at point B17 (S1 Table).

**Table 2:** Information of each area and the results of conventional PCR examinations with the primer pairs YFP-F & R (finless porpoise; *Neophocaena* sp.) and Slo4-F & R (oceanic dolphins).

Area	Date	Location	Number of samples	Number of Positive of *Neophocaena* sp.	Ratio of *Neophocaena* sp. positive (%)	Number of Positive of oceanic dolphins	Ratio of oceanic dolphins positive (%)	The number of samples positive for each oceanic dolphin species			
								Common dolphin	Indo-Pacific bottlenose dolphin	Orca	Spinner dolphin
A	2022 July	Jeju beach	39	37	95	16	41	5	6	5	1
B	2023 July	Jeju beach	19	9	47	3	16	2	1	1	0
C	2022 Jun	Chuja Islands	13	9	69	6	46	0	3	4	0
D	2023 Jun	Southern Sea & Jeju Sea	24	11	46	8	33	2	3	3	0
E	2022 May	The Yellow Sea	18	12	67	9	50	5	0	4	0
F	2023 May	The Yellow Sea	20	12	60	8	40	3	0	5	0
G	2023 Jun	Southern sea of Jeju Island	6	4	67	0	0	0	0	0	0
**Total**			**139**	**94**	**68**	**50**	**36**	**17**	**13**	**19**	**1**

Area A consisted of seawater from the Jeju coastline collected in July 2022, where 37 of 39 samples tested positive (95%). Area B included seawater from the Jeju coastline collected in July 2023, with 9 out of 19 samples showing positive reactions (47%). Area C contained seawater from the Chuja Islands collected in June 2022, where 9 out of 13 samples tested positive (69%). Area D included seawater from the southern region and Jeju, collected in June 2023, with 11 of the 24 samples showing positive reactions (46%). Area E consisted of seawater from the Yellow Sea collected in May 2022, where 12 of 18 samples tested positive (67%). Area F included seawater from the Yellow Sea collected in May 2023, with 12 of 20 samples showing positive reactions (60%). Finally, Area G consisted of seawater from the southern region of Jeju Island collected in June 2023, where four of six samples tested positive (67%).

### Conventional and real-time PCR examination of oceanic dolphin microsatellite Slo4 sequence

The results of conventional PCR for detecting oceanic dolphin microsatellite Slo4 gene sequence in all eDNA samples are summarized in Table 2, S2 Table, and Fig 2. Of the 139 eDNA samples, microsatellite Slo4 gene sequences of oceanic dolphins were detected in 50 (36%; Table 2). During the annotation process, Atlantic cetacean species not distributed in the Pacific Ocean were excluded such as white-beaked dolphin (*Lagenorhynchus albirostris*), Sowerby’s beaked whale (*Mesoplodon bidens*), and northern bottlenose whale (*Hyperoodon ampullatus*), and only species that have possibility to be found in Korea were selected for further analysis. Out of 139 eDNA samples, DNA sequences of common dolphins were detected in 17 samples, IPBD in 13 samples, orcas in 19 samples, and spinner dolphins in a sample. Most positive samples had high values, with a query coverage of over 96% and an identity of over 97% (S2 Table). In all positive samples, the eDNA concentration of the oceanic dolphins ranged from 10^3^ to 10^6^ copies/µL, with particularly high concentrations of 1.01 × 10^5^ copies/µL at point C03, 1.64 × 10^5^ copies/µL at point C04, 3.66 × 10^5^ copies/µL at point C08, 1.23 × 10^5^ copies/µL at point C12, 1.38 × 10^6^ copies/µL at point E12, 1.96 × 10^6^ copies/µL at point F02, 2.25 × 10^5^ copies/µL at point F05, and 1.27 × 10^5^ copies/µL at point F19 (S2 Table).

Of the 39 seawater samples from Area A, 16 showed positive reactions (41%). The detections included eDNA of five common dolphin, six IPBDs, five orcas, and one spinner dolphin. Of the 19 seawater samples from Area B, three showed positive reactions (16%), including eDNA detections of two common dolphins, one IPBD, and one orca. Among the 13 seawater samples from Area C, six tested positive (46%). eDNA of three IPBDs and four orcas were detected. In the 24 seawater samples from Area D, eight showed positive reactions (33%), with eDNA of two common dolphins, three IPBD and three orca detected. Of the 18 seawater samples from Area E, nine tested positive (50%), including five common dolphins and four orcas. In the 20 seawater samples from Area F, eight showed positive reactions (40%), with the eDNA detection of three common dolphins and five orcas. All six seawater samples from Area G tested negative (0%).

### Distribution analysis of cetacean eDNA in Korean Seas

In July 2022, among the 39 seawater samples collected from Area A (Jeju Island coastline), eDNA of the finless porpoises (*Neophocaena* sp.) was detected at all points except A10 and A16 (Fig 2A and S1 Table). The IPBD resides close to the coast of Jeju Island, and eDNA of this species was detected at A02, A26, A33, A34, A36, and A39 (Fig 2A and S2 Table). Additionally, eDNA of orcas was detected at A08, A10, A14, A15, and A18, whereas eDNA of common dolphins was detected at A05, A20, A21, A26, and A37. eDNA of spinner dolphin was detected at A32.

In contrast, among the 19 samples collected from Area B (Jeju Island coastline) in July 2023, eDNA from the finless porpoises was detected at the points B01, B02, B08, B11, B14, B16, and B17 (Fig 2B and S1 Table), whereas eDNA from the IPBD was detected at B03 (Fig 2B and S2 Table). The eDNA of orcas was detected at B08, while that of common dolphins was detected at B08 and B11.

In June 2022, among the 13 seawater samples collected from Area C (Chuja Islands in Jeju waters), the eDNA of the finless porpoises was detected at points C01, C03, C05, C06, C07, C10, C11, C12, and C13 (Fig 2C and S1 Table), whereas the eDNA of the IPBD was detected at C03, C08, and C09 (Fig 2C and S2 Table). The eDNA of orcas was detected at C03, C04, C11, and C12.

In June 2023, among the 24 samples collected from Area D (Jeju Sea and Southern Sea of Korea), eDNA of the finless porpoises was detected at points D01, D04, D06, D07, D08, D09, D10, D15, D20, D21, and D23 (Fig 2D and S1 Table). The eDNA of the IPBDs was detected at points D03, D05, and D20, whereas the eDNA of orcas was detected at D10, D13, and D18, and the eDNA of common dolphins was detected at D15 and D17 (Fig 2D and S2 Table).

In May 2022, among the seawater samples collected from Area E (the Yellow Sea), the eDNA of the finless porpoises was detected at points E01, E02, E03, E04, E05, E06, E07, E08, E15, E16, E17, and E18 (Fig 2E and S1 Table). eDNA of common dolphins was detected at E01, E02, E03, E04, and E18; and eDNA of orcas was detected at E07, E08, E12, and E13 (Fig 2E and S2 Table).

In May 2023, among the 20 samples collected from Area F (the Yellow Sea), eDNA of the finless porpoises was detected at points F01, F02, F03, F05, F07, F08, F11, F12, F13, F14, F19, and F20 (Fig 2F and S1 Table). The eDNA of common dolphins was detected at F02, F05, and F12; and eDNA of orcas was detected at F03, F04, F15, F18, and F19 (Fig 2F and S2 Table).

In June 2023, among the seawater samples collected from Area G (southern waters of Jeju Island), eDNA of the finless porpoises was detected only at points G01, G02, G03, and G04, whereas eDNA of the oceanic dolphins was not detected (Fig 2G and S1 Table).

### Phylogenetic analysis

**Fig 3:**
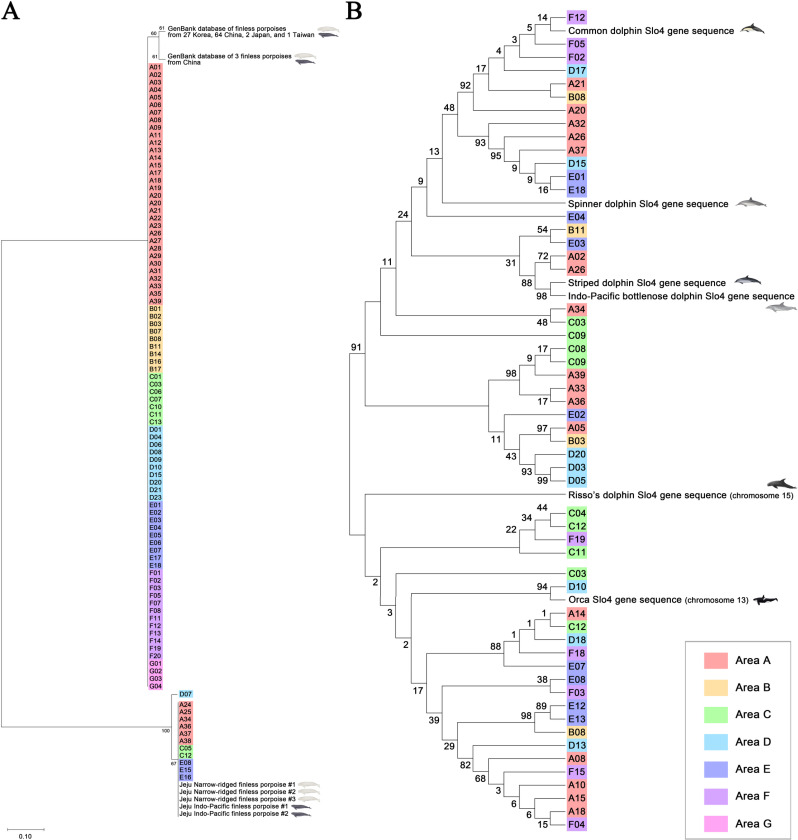
Maximum-likelihood trees of eDNA. Samples from each area are indicated in different colors. Area A is represented in light red, area B in light orange, area C in light green, area D in sky blue, area E in blue, area F in purple, and area G in pink. The sequences of the positive controls and the GenBank database are represented by cetacean drawings illustrated by S.B.L. **A** Phylogenetic tree of *Neophocaena* sp. mtDNA D-loop gene partial sequences. eDNA sequences were analyzed with sequences from Jeju finless porpoises and GenBank sequences from Korean peninsula, China, and Japan. The tree was visualized with branch lengths included. The lengths of the branches represent the evolutionary distances between the clusters. **B** Phylogenetic tree of Delphinidae microsatellite Slo4 gene sequences. eDNA sequences were analyzed with Slo4 sequences of IPBD, common dolphin, spinner dolphin, striped dolphin, and chromosomes of Risso’s dolphin and orca. The tree was visualized with topology only.

The phylogenetic analysis of *Neophocaena* sp. eDNA revealed that the partial mtDNA D-loop gene sequences from samples A24, A25, A34, A37, A38, C05, C12, D07, E08, E15, and E16 are genetically close to those of finless porpoises stranded on Jeju Island (Fig 3A). Meanwhile, the sequences detected from 32 samples in area A, 9 samples in area B, 7 samples in area C, 10 samples in area D, 9 samples in area E, 12 samples in area F, and 4 samples in area G showed a close genetic distance to the sequences of *Neophocaena* sp. from Korean Peninsula, China, Japan, and Taiwan.

The phylogenetic analysis of Delphinidae microsatellite Slo4 gene sequences indicated a close genetic relationship between the Slo4 gene sequences of common dolphin and spinner dolphin, while the sequences of striped dolphin and IPBD are genetically close (Fig 3B). The sequences from samples A20, A21, A26, A32, A37, B08, D15, D17, E01, E04, E18, F02, F05, and F12 showed a close relationship with the microsatellite Slo4 gene sequences of common dolphin and spinner dolphin. The sequences from samples A02, A05, A26, A34, B03, B11, C03, C08, C09, D03, D05, D20, E02, and E03 showed a close relationship with the microsatellite Slo4 gene sequences of IPBD and striped dolphin. The sequences from samples A08, A10, A14, A15, A18, B08, C12, D13, D18, E07, E08, E12, E13, F03, F04, F15, and F18 showed a close relationship with the microsatellite Slo4 gene sequence of orca.

### Haplotype network analysis

**Fig 4:**
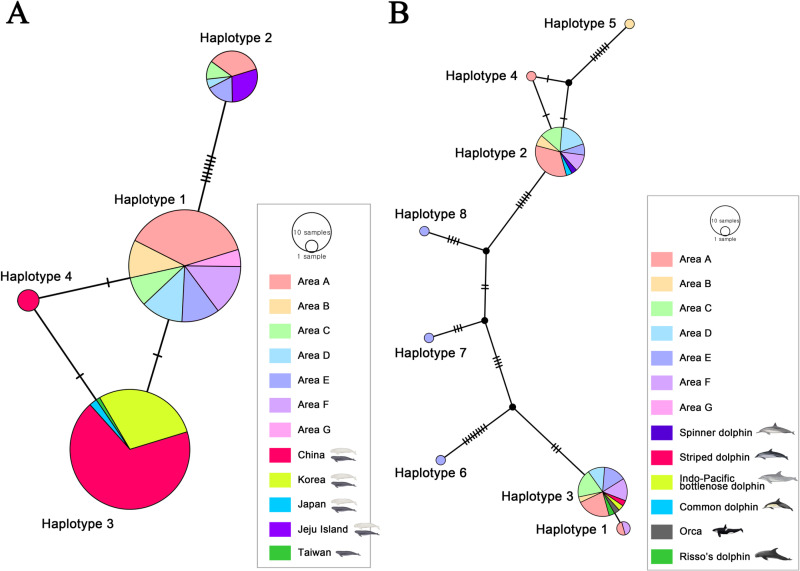
Inferred genealogical relationship among the haplotypes of cetacean eDNA based on a Templeton-Crandall-Sing (TCS) network. Samples from each area are indicated in different colors. Area A is represented in light red, area B in light orange, area C in light green, area D in sky blue, area E in blue, area F in purple, and area G in pink. The sequences of the positive controls and the GenBank database are represented by different colors and cetacean drawings illustrated by S.B.L. **A** Haplotype network of mtDNA D-loop gene partial sequences in finless porpoises. **B** Haplotype network of microsatellite Slo4 gene sequences in Delphinidae.

The visualization of the haplotype network revealed that there are four haplotypes included in the mtDNA D-loop partial gene sequences of *Neophocaena* sp. in this study (Fig 4A). Eighty-two eDNA sequences contained haplotype 1, while the sequences from samples A24, A25, A34, A36, A37, A38, C05, C12, D07, E08, E15, and E16 possessed haplotype 2 same as the sequences amplified from the tissues of finless porpoises in Jeju Island. Haplotype 3 was found in 94 sequences of finless porpoises from the Korean peninsula, China, Japan, and Taiwan, while haplotype 4 was included in three rodml sequences of finless porpoises from China. Haplotype 1, 3, and 4 showed close relationships in the TCS network result.

The visualization of the haplotype network showed that eight haplotypes included in the microsatellite Slo4 gene sequences of Delphinidae (Fig 4B). Haplotype 1 was found in eDNA samples A02 and F18. Haplotype 2 was included in the Slo4 gene sequences of common dolphin, spinner dolphin, eDNA samples A05, A20, A26, A32, A33, A34, A36, A37, A39, B03, B08, C03, C08, C09, D03, D05, D15, D17, D20, E01, E18, F02, F05, and F12. The Slo4 sequences of IPBD, orca, Risso’s dolphin, and striped dolphin included haplotype 3, which was also found in the sequences of eDNA samples A08, A10, A14, A15, A18, A26, B08, C03, C04, C11, C12, D10, D13, D18, E07, E08, E12, E13, F03, F04, F15, and F19. Haplotype 4, 5, 6, 7, and 8 were respectively detected in eDNA samples A21, B11, E02, E03, and E04. Haplotype 1 and 3, as well as haplotype 2 and 4, showed close relationships, while haplotypes 5, 6, 7, and 8 showed independent relationships in the TCS network result.

### Cetacean sightings around Jeju Island

**Fig 5:**
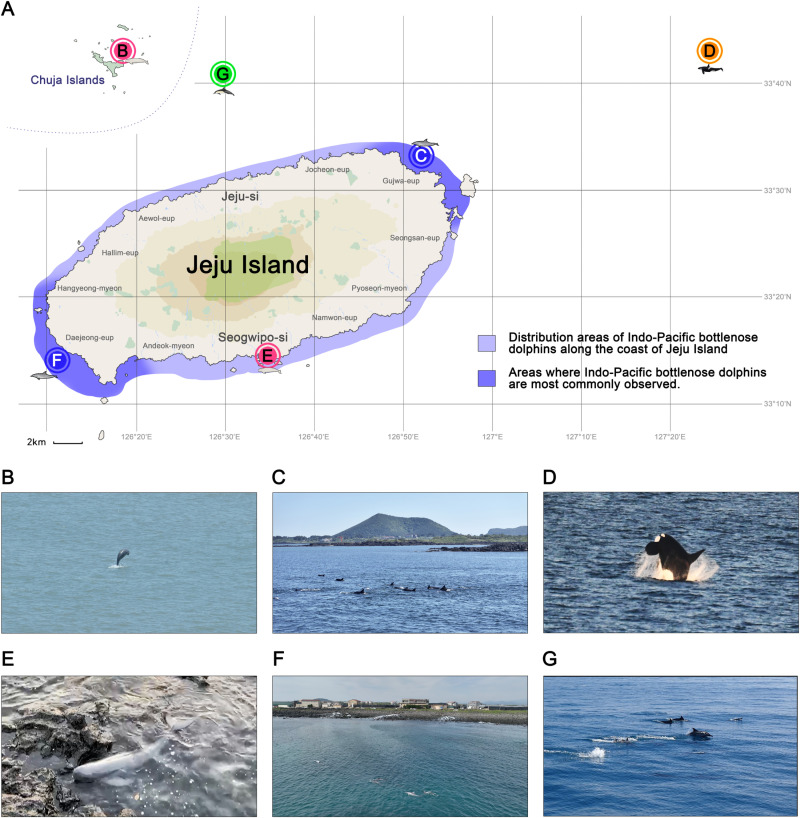
Records of cetacean sightings around Jeju Island. All cetacean photographs were taken and provided by Byung Yeop Kim, and the illustration was created by Sung Bin Lee. **A** A map of Jeju Island with records of cetacean discovery points. The distribution of Indo-Pacific bottlenose dolphins (IPBD) is marked in blue, with darker areas indicating regions where IPBD are most frequently found. **B** On June 3, 2023, a narrow-ridged finless porpoise (NRFP) was found at Chuja Islands (33°57′45.4′′N 126°19′31.4′′E). The Chuja Islands are the easiest area to spot NRFP in the waters around Jeju Island. **C** On June 4, 2024, a school of IPBD was observed in the Gujwa-eup area (33°33′06.3′′N 126°51′41.0′′E). **D** An orca was discovered on July 26, 2022, in the eastern waters of Jeju Island (33°44′16.0′′N 127°24′36.4′′E). **E** On October 11, 2020, an NRFP that had stranded was rescued along the coast of Seogwipo-si (33°14′25.2′′N 126°32′13.6′′E). **F** On April 18, 2024, a school of IPBD was observed in the Daejeong-eup area (33°12′42.5′′N 126°14′31.9′′E). **G** On February 27, 2023, a school of common dolphins was observed in the northern waters of Jeju Island (33°40′46.2′′N 126°29′07.4′′E).

The target species were observed in the waters around Jeju Island, and the observations for each species have been illustrated on a map of Jeju Island (Fig 5A). On June 3, 2023, a NRFP was found at Chuja Islands seawater during shipboard observations (33°57′45.4′′N 126°19′31.4′′E; Fig 5B). The Chuja Islands are located in the Southern Sea of Korea, between the Korean Peninsula and Jeju Island, and are geographically included in Jeju waters. The islands are the easiest areas in which NRFP can be detected in the waters around Jeju Island. A school of IPBDs was observed in the Gujwa-eup area (33°33′06.3′′N 126°51′41.0′′E; Fig 5C) on June 4, 2024 during shipboard observations. IPBDs were primarily distributed along the coast of Jeju Island and were found in all coastal areas of Jeju Island. Among these, the Gujwa-eup and Daejeong-eup areas were zones where IPBDs spent the most time engaged in activities, such as foraging, making them the areas where IPBDs were frequently observed (Fig 5A). An orca was discovered on July 26, 2022, in the eastern waters of Jeju Island (33°44′16.0′′N 127°24′36.4′′E; Fig 5D) during shipboard observations. Orcas are rarely observed in Korean waters and tend to prefer coastal seawater over pelagic environments, indicating that Korean waters are clearly part of the orca’s activity range in the East Asian area. On October 11, 2020, an NRFP that had stranded was found shore-based observations and rescued along the coast of Seogwipo-si (33°14′25.2′′N 126°32′13.6′′E; Fig 5E). This is a rare and exceptional case in which a NRFP was found alive in the waters very close to Jeju Island. A school of IPBD was observed in the Daejeong-eup area (33°12′42.5′′N 126°14′31.9′′E; Fig 5F) on April 18, 2024 using a drone. On February 27, 2023, a school of common dolphins was observed in the northern waters of Jeju Island (33°40′46.2′′N 126°29′07.4′′E; Fig 5G) during shipboard observations. Common dolphins are difficult to observe in waters very close to Jeju Island; however, they are commonly found in the northern waters of Jeju and along the southern coast of Korea.

## Discussion

The NRFP has been registered as an internationally endangered species by the IUCN since 2017 because of a drastic population decline caused by bycatch [7–9]. Additionally, the target species of this study, including the NRFP, IPBD, orca, and common dolphin, were designated as Marine Organisms under Protection by the Ministry of Oceans and Fisheries of the Republic of Korea [56]. To conserve populations of these cetacean species, research on their ecology, physiology, and veterinary pathology must be actively conducted, with distribution and population surveys serving as the most fundamental basis. Traditional cetacean vessel surveys are conducted annually in Korean waters; however, methods, such as vessel surveys, scuba diving, and usage of drones, have several limitations [27,28].

For the dominant species in Korean waters, the NRFP, its shy nature, and anatomical features, such as the absence of a dorsal fin, make it difficult to observe at the water surface [57,58]. The Indo-Pacific finless porpoise, which has been rarely observed in Korean waters and has recently been occasionally found in the waters around Jeju Island, shares anatomically similar characteristics with the NRFP, making expert opinions crucial for distinguishing between these two species [57,59]. Vessel surveys rely heavily on experts skilled in cetacean species identification, which is a significant limitation of this study [57,60]. They are also significantly affected by weather conditions and surveys cannot be conducted in inaccessible areas [61]. Budget consideration owing to economic losses from vessel operations is also an important factor [62].

Unlike traditional methods, eDNA-based distribution surveys can be conducted in virtually all marine habitats [63–65]. This study confirms that high concentrations of eDNA from cetacean species can be detected even in shallow coastal waters. eDNA research requires less effort and expertise for sampling, thus providing a temporal advantage [66,67]. Since eDNA research began in the early 2000s, its usefulness for monitoring species with unclear whole-genome sequences and DNA barcodes has remained limited [68]. In this study, the mtDNA D-loop gene partial sequence of the NRFP amplified using the YFP-F & R primers could not be distinguished from that of the genetically similar Indo-Pacific finless porpoise. Although the Indo-Pacific finless porpoise does not officially inhabit Korean waters [57], it has been occasionally found in Jeju recently [59]. As the rise in sea temperatures due to global warming may also affect the distribution of the Indo-Pacific finless porpoise in Korea, it is necessary to develop primers that can distinguish the genetic characteristics of the two finless porpoise species in the future to enable more efficient eDNA monitoring.

According to previous research on the eDNA of the Yangtze finless porpoise (*N. asiaeorientalis asiaeorientalis*), higher concentrations of eDNA were detected in samples collected during summer than in those collected during winter [30]. The Yangtze finless porpoise is classified as a subspecies distinct from the NRFP (*N. asiaeorientalis sunameri*) found in Korean waters but shares genetically similar characteristics [57,69]. Stow net fishing activities are actively conducted in the southern sea and Jeju waters of Korea during winter [70]. The prey of various coastal *Odontoceti* species, including the NRFP and oceanic dolphins, overlap with the target species of stow nets, leading to bycatch and increased incidences of suffocation during winter [71,72]. Additional investigation is needed, however to exclude the possibility that cetacean carcasses that died from asphyxiation may result in false positives for eDNA, sampling for this study was primarily conducted from late spring to summer. Although a previous study on eDNA of crayfish have indicated that crayfish carcasses do not produce detectable eDNA, it remains uncertain how this applies to eDNA studies of cetaceans with significant biomass [73].

The detection of eDNA in marine ecosystems has numerous limitations compared to that in freshwater systems. The biomass-to-water ratio is low in marine environments [74,75], and factors, such as the influence of currents on the movement and degradation of eDNA [76] and the impact of salinity on eDNA preservation [77], can affect eDNA detection. The detection, occurrence, and degradation of eDNA in marine environments are influenced by a variety of environmental factors and the state of eDNA (intracellular or extracellular), which has led to ongoing debate in the academic community regarding the degradation rates of eDNA in seawater. However, it has been suggested that the DNA degradation rate in seawater is approximately 10–48 h, which is significantly higher than that in freshwater [28,78,79]. This implies that the eDNA in seawater has a lower probability of dispersal over long distances, breaks down more rapidly, and is present at lower concentrations [28]. However, the results of this study show, for the first time, that eDNA from various cetacean species exists at high densities in Korean seawater.

The average current velocity in the sampling areas of the Yellow Sea, South Sea, and Jeju waters was 0.2 m/s. Based on previous research, it can be estimated that eDNA can move between approximately 8 and 120 km before complete degradation [28]. The membrane filter used in this study had a pore size of 0.45 µm; therefore, considering the size of DNA molecules, the detected eDNA was likely to be from skin cells, intestinal cells, and other sources [80]. However, various factors, such as seawater temperature, changes in regional currents, wind speed, typhoons, and the movement of species, may have influenced the degradation and concentration of eDNA, leading to slightly different eDNA detection results in this study for 2022 and 2023.

In July 2022, various cetacean species, including NRFP and IPBD, showed positive reactions off the coast of Jeju Island (Fig 2A), whereas in July 2023, the rate of positive reactions was low (Fig 2B). Additionally, points E09–E11 showed negative reactions in May 2022 (Fig 2E), whereas locations F11–F14 showed positive reactions in May 2023 (Fig 2F). As prey species migrate, cetacean species may also move, and various other factors may influence their distribution [81,82]. Therefore, further monitoring and research on cetacean eDNA is necessary.

The presence of NRFP in the waters around Jeju Island is the subject of ongoing debate. However, over the past 4 years, 34 carcasses of NRFP and three carcasses of Indo-Pacific finless porpoises have been discovered along the coast of Jeju. These sightings, including the observations of NRFP in the waters surrounding Chuja-do, indicate their potential presence (Figs 5B and 5E). Additionally, the eDNA detection results from this study support the possibility of their habitat in Jeju waters (Figs 2A, 2B, 2C, 2D, and 2G).

The coastal waters of Jeju host approximately 120 IPBDs, which may hinder access for other cetacean species [32]. It is speculated that NRFPs are distributed in waters relatively distant from Jeju because of competition for food. The eDNA of finless porpoises (*Neophocaena* sp.) detected along the coast of Jeju Island is considered to originate from cells, feces, and other materials from porpoises in the offshore areas of Jeju Island. Among the points where the eDNA of the finless porpoises was detected, those with high concentrations were primarily located along the northern coast of Jeju, relatively close to Chuja-do (A01, A32, A35, A36, and B02) (S1 Table). Because the presence of NRFP has been confirmed multiple times in the waters around Chuja-do, it is likely that they are distributed between Chuja-do and Jeju. However, it is essential to consider that eDNA concentrations can be influenced by various factors, including ocean currents. The southern waters of Jeju Island have a deep-sea environment that is unsuitable for distribution of finless porpoises (*Neophocaena* sp.), yet eDNA of finless porpoises was detected in this location. It is presumed that this eDNA was transported along the currents from the far southwest sea (Figs 2A and 2B) and was detected throughout the coast of Jeju Island.

IPBDs inhabit the entire coastal area of Jeju Island, with frequent sightings, particularly around Daejeong-eup and Gujwa-eup (Figs 5A, 5C, and 5F) [33]. In 2022, eDNA was detected in different coastal areas (Fig 2A), whereas in 2023, eDNA was detected at the point B03 and D20 (Figs 2B and 2D). As eDNA degrades rapidly in seawater, the analysis results can vary depending on where these species are distributed and the areas through which they pass on the day of sampling. In the Yellow Sea area, eDNA of IPBD was not detected, but it has been detected near the waters of Chuja Island (Figs 2C and 2D). The DNA sequences of the IPBDs generally showed high identity in the annotation results (S2 Table). This suggests that there may be other small groups of IPBDs distributed in Korean seas outside of Jeju.

The orca sequences showed high query coverage and identity values at most points (S2 Table). Since East Asian orca pods could be distributed across all regions of the Korean seas, eDNA for orcas was detected in all areas except for G (Fig 2). In fact, orcas were rarely observed in Jeju waters (Fig 5D), and the eDNA results suggest that they may also be distributed in the Yellow Sea and southern waters of Korea.

For the common dolphin, eDNA was detected in Jeju waters, except around the Chuja-do area, but was repeatedly found off the northern and western coast of Jeju and the southern coast of Chuja-do (Figs 2A, 2B, 2C, and 2D). This species was rarely observed in Jeju waters (Fig 5G), and based on the eDNA results, it was estimated to primarily inhabit the northern and western waters of Jeju. Furthermore, in May 2022 and 2023, large numbers of eDNA sequences were detected in the northern Yellow Sea (Figs 2E and 2F).

In previous studies, despite the fact that the waters of Korea are included in the habitat distribution of striped dolphins (*Stenella coeruleoalba*) and spinner dolphins (*Stenella longirostris*) [83,84], these dolphins are species of oceanic dolphin that have not been recorded in Korea. However, in the present study, the eDNA sequence of spinner dolphins was detected at point A32 (Fig 2A and S2 Table). Common dolphins and spinner dolphins have a close relationship on the phylogenetic tree of microsatellite Slo4 and share the same haplotype, so the possibility of it being eDNA from common dolphins cannot be ruled out (Figs 3B and 4B). This result may also be a false positive. False positive detections of eDNA can occur from sources other than animals, such as sewage and wastewater, feces from other predatory animals, or dead animals [85]. The impact of these factors has not yet been clearly established in the eDNA research community, especially in studies involving cetaceans. However, query coverage and identity of the Slo4 sequence of A32 were higher with spinner dolphin’s one than common dolphin’s one (S2 Table). Although these two species have not been previously recorded in Korea, the recent increase in seawater temperature owing to global warming may have facilitated their influx into the southernmost waters of Jeju Island [86]. Further research, along with cetacean surveys, is required to better understand the distribution of these two species.

eDNA research has many advantages and has been shown to be a useful tool for distribution surveys of cetacean species in previous studies [87–89]. Although the lack of visual surveys is a limitation of this study, this study represents the first report of cetacean eDNA research conducted in the Korean seas, indicating that more diverse studies need to be conducted in the future. Additionally, there is a limitation in that sampling process was only performed in the summer based on vessel operational schedule and previous study results [30,31]. Since the primary purpose of operating the Ara-ho vessel was for student training in ship operations, there was a limitation in not being able to sample at the same locations during the sampling processes in 2022 and 2023 that aligned with the objectives of our eDNA study. Our research team made efforts to match the sampling sites as closely as possible, and thus the sampling along the coast of Jeju Island in 2022 (Fig 2A), 2023 (Fig 2B), and the Yellow Sea in 2022 (Fig 2E) and 2023 (Fig 2F) was mostly conducted in the same areas. However, for the other regions (Figs 2C, 2D, and 2G), it was challenging to sample in the same locations due to scheduling of Ara-ho’s operation. In the future, if we conduct sampling in winter in collaboration with the vessel operation team, we expect to derive more meaningful conclusions by comparing the result with this study. Additionally, we aim to conduct sampling at the same locations during both summer and winter, as well as across different years, to facilitate data comparison.

The primer pair YFP-F & R was used in this study since it demonstrated high amplification efficiency even in low concentrations of eDNA. Additionally, the mtDNA D-loop gene partial sequence of *Neophocaena* sp. detected in this study is a short sequence of 102 bp, which is convenient for eDNA detection. However, this short sequence has the limitation of being insufficient to distinguish regional haplotypes in East Asia. The mtDNA D-loop gene partial sequences of *Neophocaena* sp. downloaded from the Genbank database for Korea, China, Japan, and Taiwan also did not exhibit different haplotypes in that sequence, making regional differentiation impossible. Therefore, phylogenetic analysis and haplotype network of *Neophocaena* sp. could not be thoroughly examined. If a more diverse range of haplotypes can be included and a longer mtDNA D-loop gene partial sequence can be detected from the eDNA of finless porpoises, it would be possible to efficiently analyze the inferred genealogical relationships of eDNA collected from various regions within East Asia (e.g., Yellow Sea of Korea and China, Yangtze River, Jeju Island, Omura Bay, and Tokyo Bay in Japan) similarly to previous studies [5].

While the microsatellite Slo4 gene sequence of Delphinidae is longer, ranging from 212 to 226 bp, and exhibits a variety of haplotypes, making it suitable for constructing phylogenetic analysis and haplotype networks using eDNA. However, unlike the mtDNA of finless porpoises, there has been no research on the microsatellite Slo4 gene sequence of Delphinidae species within East Asia, resulting in a lack of sufficient databases for comparing regional sequences. In this study, we detected the microsatellite Slo4 gene sequence from tissue samples of IPBD and common dolphins and added it to the GenBank database. Future research on the microsatellites of oceanic dolphins such as orca, Risso’s dolphin, and Pacific white-sided dolphin that inhabit Korean waters will enable the analysis of inferred genealogical relationships by region within Korea using eDNA.

The Korean Sea is home to various cetacean species, including NRFPs, oceanic dolphins, baleen whales, and beaked whales [6,10]. Based on this study, there is a need to expand research on a wider variety of cetacean species through eDNA analysis and to extend the research scope to include marine animals, such as sharks, sea turtles, and fish. Although we could not undertake an extensive study owing to budget constraints and the inability to invite experts, conducting cetacean eDNA research in conjunction with cetacean visual surveys is expected to yield more efficient and practical results than eDNA stand-alone experiment. We hope that this study serves as a starting point and a baseline for eDNA research on marine animals in Korea.

## Supporting information

S1 Table The amplified eDNA results of the conventional PCR and qPCR examinations with primers YFP-F & R. Each sample was collected at the specified date along with its latitude and longitude. In cases where sequences were amplified from the samples using conventional PCR, sequencing and annotation were performed to investigate the most relevant organism. Query cover indicates the extent to which the target sequence encompasses the query sequence, expressed as a percentage, while percent identity represents the similarity of the matching portions between the target sequence and the query sequence, also expressed as a percentage. The concentration of eDNA was measured using qPCR.
(DOCX)

S2 Table The amplified eDNA results of the conventional PCR and qPCR examinations with primers Slo4-F & R. Each sample was collected at the specified date along with its latitude and longitude. In cases where sequences were amplified from the samples using conventional PCR, sequencing and annotation were performed to investigate the most relevant organism. Query cover indicates the extent to which the target sequence encompasses the query sequence, expressed as a percentage, while percent identity represents the similarity of the matching portions between the target sequence and the query sequence, also expressed as a percentage. The concentration of eDNA was measured using qPCR.
(DOCX)
