# Supplementary material for: Distribution analysis of the finless porpoises (Neophocaena sp.) and oceanic dolphins (Delphinidae) in the Korean Sea using environmental DNA
Source: PLoS One. 2025 May 16;20(5):e0322148. doi: 10.1371/journal.pone.0322148 (PMC12084060; doi:10.1371/journal.pone.0322148)
Supplement: S1 Table — Each sample was collected at the specified date along with its latitude and longitude. In cases where sequences were amplified from the samples using conventional PCR, sequencing and annotation were performed to investigate the most relevant organism. Query cover indicates the extent to which the target sequence encompasses the query sequence, expressed as a percentage, while percent identity represents the similarity of the matching portions between the target sequence and the query sequence, also expressed as a percentage. The concentration of eDNA was measured using qPCR. (DOCX) [file pone.0322148.s001.docx]

**S1 Table.** The amplified eDNA results of the conventional PCR and qPCR examinations with primers YFP-F & R. Each sample was collected at the specified date along with its latitude and longitude. In cases where sequences were amplified from the samples using conventional PCR, sequencing and annotation were performed to investigate the most relevant organism. Query cover indicates the extent to which the target sequence encompasses the query sequence, expressed as a percentage, while percent identity represents the similarity of the matching portions between the target sequence and the query sequence, also expressed as a percentage. The concentration of eDNA was measured using qPCR.

| **No.** | **The date of sampling** | **Latitude** | **Longitude** | **Amplified sequence**  **(5’ – 3’)** | **Related organism** | **Query cover**  **(%)** | **Percentidentity**  **(%)** | **Concentration of eDNA (copies / µl)** |
| --- | --- | --- | --- | --- | --- | --- | --- | --- |
| A01 | 25-July-22 | 33°26'12.8"N | 126°16'17.8"E | AGCTTAATCACCATGCCGCGTGAAACCAGCAACCCGCTCGGCAGGGATCCCTCTTCTCGCACCGGGCCCATAGCTTGTGGGGGTAGCTAAATAATGATCTA | *Neophocaena asiaeorientalis* or *N. phocaenoides* | 99 | 99 | 1.55 × 10^4^ |
| A02 | 25-July-22 | 33°24'31.7"N | 126°15'13.3"E | AGCTTAATCACCATGCCGCGTGAAACCAGCAACCCGCTCGGCAGGGATCCCTCTTCTCGCACCGGGCCCATAGCTTGTGGGGGTAGCTAAATAATGATCTA | *Neophocaena asiaeorientalis* or *N. phocaenoides* | 99 | 99 | 6.61 × 10^3^ |
| A03 | 25-July-22 | 33°39'13.0"N | 126°22'99.9"E | AGCTTAATCACCATGCCGCGTGAAACCAGCAACCCGCTCGGCAGGGATCCCTCTTCTCGCACCGGGCCCATAGCTTGTGGGGGTAGCTAAATAATGATCTA | *Neophocaena asiaeorientalis* or *N. phocaenoides* | 99 | 99 | 7.37 × 10^4^ |
| A04 | 25-July-22 | 33°21'44.3"N | 126°11'24.4"E | AGCTTAATCACCATGCCGCGTGAAACCAGCAACCCGCTCGGCAGGGATCCCTCTTCTCGCACCGGGCCCATAGCTTGTGGGGGTAGCTAAATAATGATCTA | *Neophocaena asiaeorientalis* or *N. phocaenoides* | 99 | 99 | 1.0 × 10^4^ |
| A05 | 25-July-22 | 33°21'13.7"N | 126°10'54.5"E | AGCTTAATCACCATGCCGCGTGGAACCAGCAACCCGCTCGGCAGGGATCCCTCTTCTCGCACCGGGCCCATAGCTTGTGGGGGTAGCTAAATAATGATCTA | *Neophocaena asiaeorientalis* or *N. phocaenoides* | 99 | 98 | 8.39 × 10^3^ |
| A06 | 25-July-22 | 33°18'32.0"N | 126°09'52.9"E | AGCTTAATCACCATGGCGCGTGAAACCAGCAACCCGCTCGGCAGGGATCCCTCTTCTCGCACCGGGCCCATAGCTTGTGGGGGTAGCTAAATAATGATCTA | *Neophocaena asiaeorientalis* or *N. phocaenoides* | 99 | 98 | 8.47 × 10^3^ |
| A07 | 25-July-22 | 33°16'48.0"N | 126°10'10.2"E | AGCTTAATCACCATGCCGCGTGAAACCAGCAACCCGCTCGGCAGGGATCCCTCTTCTCGCACCGGGCCCATAGCTTGTGGGGGTAGCTAAATAATGATCTA | *Neophocaena asiaeorientalis* or *N. phocaenoides* | 99 | 99 | 7.78 × 10^3^ |
| A08 | 25-July-22 | 33°15'18.0"N | 126°11'32.6"E | AGCTTAATCACCATGCCGCGTGAAACCAGCAACCCGCTCGGCAGGGATCCCTCTTCTCGCACCGGGCCCATAGCTTGTGGGGGTAGCTAAATAATGATCTA | *Neophocaena asiaeorientalis* or *N. phocaenoides* | 99 | 99 | 9.83 × 10^3^ |
| A09 | 26-July-22 | 33°14'35.2"N | 126°12'43.2"E | CCGGGGGTGCGTGAATCCTGCAACCCGCTCGGCAGGGATCCCTCTTCTCGCACCGGGCCCATAGCTTGTGGGGGTAGCTAAATAATGATCTA | *Neophocaena asiaeorientalis* or *N. phocaenoides* | 90 | 96.39 | 1.13 × 10^4^ |
| A10 | 26-July-22 | 33°14'17.5"N | 126°13'43.3"E |  |  |  |  |  |
| A11 | 26-July-22 | 33°13'53.8"N | 126°14'27.6"E | AGCTTAATCACCATGCCGCGTGAAACCAGCAACCCGCTCGGCAGGGATCCCTCTTCTCGCACCGGGCCCATAGCTTGTGGGGGTAGCTAAATAATGATCTA | *Neophocaena asiaeorientalis* or *N. phocaenoides* | 99 | 99 | 5.28 × 10^3^ |
| A12 | 26-July-22 | 33°13'29.6"N | 126°17'57.8"E | AGCTTAATCACCATGCCGCGTGAAACCAGCAACCCGCTCGGCAGGGATCCCTCTTCTCGCACCGGGCCCATAGCTTGTGGGGGTAGCTAAATAATGATCTA | *Neophocaena asiaeorientalis* or *N. phocaenoides* | 99 | 99 | 9.38 × 10^3^ |
| A13 | 26-July-22 | 33°14'26.5"N | 126°20'01.0"E | AGCTTAATCACCATGCCGCGTGAAACCAGCAACCCGCTCGGCAGGGATCCCTCTTCTCGCACCGGGCCCATAGCTTGTGGGGGTAGCTAAATAATGATCTA | *Neophocaena asiaeorientalis* or *N. phocaenoides* | 99 | 99 | 9.84 × 10^3^ |
| A14 | 26-July-22 | 33°14'39.5"N | 126°24'50.8"E | AGCTTAATCACCATGCCGCGTGAAACCAGCAACCCGCTCGGCAGGGATCCCTCTTCTCGCACCGGGCCCATAGCTTGTGGGGGTAGCTAAATAATGATCTA | *Neophocaena asiaeorientalis* or *N. phocaenoides* | 99 | 99 | 9.86 × 10^3^ |
| A15 | 26-July-22 | 33°14'17.5"N | 126°26'21.8"E | AGCTTAATCACCATGCCGCGTGAAACCAGCAACCCGCTCGGCAGGGATCCCTCTTCTCGCACCGGGCCCATAGCTTGTGGGGGTAGCTAAATAATGATCTA | *Neophocaena asiaeorientalis* or *N. phocaenoides* | 99 | 99 | 3.96 × 10^3^ |
| A16 | 26-July-22 | 33°13'36.8"N | 126°28'13.4"E |  |  |  |  |  |
| A17 | 27-July-22 | 33°14'40.2"N | 126°35'02.4"E | AGCTTAATCACCATGCCGCGTGAAACCAGGCAACCCGCTCGGCAGGGATCCCTCTTCTCGCACCGGGCCCATAGCTTGTGGGGGTAGCTAAATAATGATCTA | *Neophocaena asiaeorientalis* or *N. phocaenoides* | 99 | 98.02 | 8.23 × 10^3^ |
| A18 | 27-July-22 | 33°15'02.9"N | 126°37'17.8"E | AGCTTAATCACCATGCCGCGTGAAACCAGCAACCCGCTCGGCAGGGATCCCTCTTCTCGCACCGGGCCCATAGCTTGTGGGGGTAGCTAAATAATGATCT | *Neophocaena asiaeorientalis* or *N. phocaenoides* | 100 | 99 | 1.07 × 10^4^ |
| A19 | 27-July-22 | 33°16'08.0"N | 126°39'15.1"E | AGCTTAATCACCATGCCGCGTGAAACCAGCAACCCGCTCGGCAGGGATCCCTCTTCTCGCACCGGGCCCATAGCTTGTGGGGGTAGCTAAATAATGATCTA | *Neophocaena asiaeorientalis* or *N. phocaenoides* | 99 | 99 | 1.08 × 10^4^ |
| A20 | 27-July-22 | 33°16'11.3"N | 126°41'30.1"E | AGCTTAATCACCATGCCGCGTGAAACCAGCAACCCGCTCGGCAGGGATCCCTCTTCTCGCACCGGGCCCATAGCTTGTGGGGGTAGCTAAATAATGATCTA | *Neophocaena asiaeorientalis* or *N. phocaenoides* | 99 | 99 | 1.42 × 10^4^ |
| A21 | 27-July-22 | 33°17'15.0"N | 126°45'14.8"E | AGCTTAATCACCATGCCGCGTGAAACCAGCAACCCGCTCGGCAGGGATCCCTCTTCTCGCACCGGGCCCATAGCTTGTGGGGGTAGCTAAATAATGATCTA | *Neophocaena asiaeorientalis* or *N. phocaenoides* | 99 | 99 | 8.38 × 10^3^ |
| A22 | 27-July-22 | 33°18'13.7"N | 126°47'22.2"E | AGCTTAATCACCATGCCGCGTGAAACCAGCAACCCGCTCGGCAGGGATCCCTCTTCTCGCACCGGGCCCATAGCTTGTGGGGGTAGCTAAATAATGATCTA | *Neophocaena asiaeorientalis* or *N. phocaenoides* | 99 | 99 | 9.32 × 10^3^ |
| A23 | 27-July-22 | 33°19'21.0"N | 126°50'48.8"E | CGCTCGGCAGGGATCCCTCTTCTCGCACCGGGCCCATAGCTTGTGGGGGTAGCTAAATAATGATCT | *Neophocaena asiaeorientalis* or *N. phocaenoides* | 100 | 98.48 | 7.47 × 10^3^ |
| A24 | 27-July-22 | 33°20'57.1"N | 126°51'48.6"E | AGTGATTAAGCTCGTGATCTAATGGAGCGGCCATAGGATATAATGGTTATGAAGGGCTAGTGGACATAA | *Neophocaena asiaeorientalis* or *N. phocaenoides* | 97 | 100 | 8.57 × 10^3^ |
| A25 | 28-July-22 | 33°24'18.0"N | 126°54'18.7"E | GTGATTAAGCTCGTGATCTAATGGAGCGGCCATAGGATATAATGGTTATGAAGGGCTAGTGGACATAA | *Neophocaena asiaeorientalis* or *N. phocaenoides* | 98 | 100 | 7.36 × 10^3^ |
| A26 | 28-July-22 | 33°25'59.5"N | 126°55'45.1"E | TCACCATGCCGCGTGAAACCAGCAACCCGCTCGGCAGGGATCCCTCTTCTCGCACCGGGCCCATAGCTTGTGGGGGTAGCTAAATAATGATCAG | *Neophocaena asiaeorientalis* or *N. phocaenoides* | 97 | 98.91 | 8.53 × 10^3^ |
| A27 | 28-July-22 | 33°30'45.0"N | 126°53'54.6"E | CGAGCTTAATCACCATGCCGCGTGAAACCAGCAACCCGCTCGGCAGGGATCCCTCTTCTCGCACCGGGCCCATAGCTTGTGGGGGTAGCTAAATAATGATCT | *Neophocaena asiaeorientalis* or *N. phocaenoides* | 100 | 99.02 | 1.01 × 10^4^ |
| A28 | 28-July-22 | 33°32'01.0"N | 126°50'24.4"E | GAGCTTAATCACCATGCCGCGTGAAACCAGCAACCCGCTCGGCAGGGATCCCTCTTCTCGCACCGGGCCCATAGCTTGTGGGGGTAGCTAAATAATGATCTA | *Neophocaena asiaeorientalis* or *N. phocaenoides* | 99 | 99.01 | 8.39 × 10^3^ |
| A29 | 28-July-22 | 33°33'35.6"N | 126°48'49.7"E | AGCTTAATCACCATGCCGCGTGAAACCAGCAACCCGCTCGGCAGGGATCCCTCTTCTCGCACCGGGCCCATAGCTTGTGGGGGTAGCTAAATAATGATCT | *Neophocaena asiaeorientalis* or *N. phocaenoides* | 100 | 99 | 1.31 × 10^4^ |
| A30 | 28-July-22 | 33°33'29.5"N | 126°44'55.0"E | AGCTTAATCACCATGCCGCGTGAAACCAGCAACCCGCTCGGCAGGGATCCCTCTTCTCGCACCGGGCCCATAGCTTGTGGGGGTAGCTAAATAATGATCT | *Neophocaena asiaeorientalis* or *N. phocaenoides* | 100 | 99 | 1.36 × 10^4^ |
| A31 | 28-July-22 | 33°32'55.7"N | 126°41'02.8"E | AGCTTAATCACCATGCCGCGTGAAACCAGCAACCCGCTCGGCAGGGATCCCTCTTCTCGCACCGGGCCCATAGCTTGTGGGGGTAGCTAAATAATGATCT | *Neophocaena asiaeorientalis* or *N. phocaenoides* | 100 | 99 | 8.06 × 10^3^ |
| A32 | 28-July-22 | 33°33'11.2"N | 126°38'37.0"E | CGAGCTTAATCACCATGCCGCGTGAAACCAGCAACCCGCTCGGCAGGGATCCCTCTTCTCGCACCGGGCCCATAGCTTGTGGGGGTAGCTAAATAATGATCTA | *Neophocaena asiaeorientalis* or *N. phocaenoides* | 99 | 99.02 | 1.62 × 10^4^ |
| A33 | 29-July-22 | 33°31'32.5"N | 126°35'01.3"E | CGAGCTTAATCACCATGCCGCGTGAAACCAGCAACCCGCTCGGCAGGGATCCCTCTTCTCGCACCGGGCCCATAGCTTGTGGGGGTAGCTAAATAATGATCTA | *Neophocaena asiaeorientalis* or *N. phocaenoides* | 99 | 99.02 | 1.45 × 10^4^ |
| A34 | 29-July-22 | 33°31'36.8"N | 126°33'50.0"E | GGTGATTAAGCTCGTGATCTAATGGAGCGGCCATAGGATATAATGGTTATGAAGGGCTAGTGGACATAA | *Neophocaena asiaeorientalis* or *N. phocaenoides* | 98 | 100 | 1.19 × 10^4^ |
| A35 | 29-July-22 | 33°31'36.8"N | 126°33'50.0"E | GCTCGGCAGGGATCCCTCTTCTCGCACCGGGCCCATAGCTTGTGGGGGTAGCTAAATAATGATCTA | *Neophocaena asiaeorientalis* or *N. phocaenoides* | 98 | 98.46 | 1.7 × 10^4^ |
| A36 | 29-July-22 | 33°29'53.9"N | 126°27'11.5"E | GGTGATTAAGCTCGTGATCTAATGGAGCGGCCATAGGATATAATGGTTATGAAGGGCTAGTGGACATAA | *Neophocaena asiaeorientalis* or *N. phocaenoides* | 98 | 100 | 1.8 × 10^4^ |
| A37 | 29-July-22 | 33°29'02.8"N | 126°23'42.0"E | TGCCGAGCGGGTTGCTGGTTTCACGCGGCATGGTGATTAAGCTCGTGATCTAATGGAGCGGCCATAGGATATAATGGTTATGAAGGGCTAGTGGACATAA | *Neophocaena asiaeorientalis* or *N. phocaenoides* | 99 | 100 | 1.13 × 10^4^ |
| A38 | 29-July-22 | 33°28'21.4"N | 126°20'54.2"E | CCCTGCCGAGCGGGTTGCTGGTTTCACGCGGCATGGTGATTAAGCTCGTGATCTAATGGAGCGGCCATAGGATATAATGGTTATGAAGGGCTAGTGGACATAA | *Neophocaena asiaeorientalis* or *N. phocaenoides* | 99 | 100 | 8.45 × 10^3^ |
| A39 | 29-July-22 | 33°27'12.2"N | 126°18'25.2"E | CTTAATCACCATGCCGCGTGAAACCAGCAACCCGCTCGGCAGGGATCCCTCTTCTCGCACCGGGCCCATAGCTTGTGGGGGTAGCTAAATAATGATCTA | *Neophocaena asiaeorientalis* or *N. phocaenoides* | 98 | 98.98 | 9.57 × 10^3^ |
| B01 | 10-July-23 | 33°29'39.5"N | 126°25'57.5"E | AGCTTAATCACCATGCCGCGTGAAACCAGCAACCCGCTCGGCAGGGATCCCTCTTCTCGCACCGGGCCCATAGCTTGTGGGGGTAGCTAAATAATGATCTA | *Neophocaena asiaeorientalis* or *N. phocaenoides* | 99 | 99 | 6.29 × 10^3^ |
| B02 | 10-July-23 | 33°28'24.4"N | 126°20'59.5"E | AGCTTAATCACCATGCCGCGTGAAACCAGCAACCCGCTCGGCAGGGATCCCTCTTCTCGCACCGGGCCCATAGCTTGTGGGGGTAGCTAAATAATGATCT | *Neophocaena asiaeorientalis* or *N. phocaenoides* | 100 | 99 | 1.47 × 10^4^ |
| B03 | 10-July-23 | 33°26'13.1"N | 126°16'18.0"E | AGCTTAATCACCATGCCGCGTGAAACCAGCAACCCGCTCGGCAGGGATCCCTCTTCTCGCACCGGGCCCATAGCTTGTGGGGGTAGCTAAATAATGATCTA | *Neophocaena asiaeorientalis* or *N. phocaenoides* | 99 | 99 | 4.76 × 10^3^ |
| B04 | 10-July-23 | 33°24'10.0"N | 126°15'02.6"E |  |  |  |  |  |
| B05 | 10-July-23 | 33°21'44.0"N | 126°11'23.8"E |  |  |  |  |  |
| B06 | 10-July-23 | 33°16'42.0"N | 126°10'09.7"E |  |  |  |  |  |
| B07 | 10-July-23 | 33°15'52.1"N | 126°10'50.4"E | AGCTTAATCACCGTGCCGCGTGAAACCAGCAACCCGCTCGGCAGGGATCCCTCTTCTCGCACCGGGCCCATAGCTTGTGGGGGTAGCTAAATAATGATCT | *Neophocaena asiaeorientalis* or *N. phocaenoides* | 100 | 98 | 1.3 × 10^4^ |
| B08 | 10-July-23 | 33°14'32.5"N | 126°12'44.0"E | AGCTTAATCACCATGCCGCGTGAAACCAGCAACCCGCTCGGCAGGGATCCCTCTTCTCGCACCGGGCCCATAGCTTGTGGGGGTAGCTAAATAATGATCTA | *Neophocaena asiaeorientalis* or *N. phocaenoides* | 99 | 99 | 9.86 × 10^3^ |
| B09 | 10-July-23 | 33°14'17.7"N | 126°13'42.5"E |  |  |  |  |  |
| B10 | 11-July-23 | 33°14'39.4"N | 126°24'50.3"E |  |  |  |  |  |
| B11 | 11-July-23 | 33°16'10.7"N | 126°40'23.5"E | AGCTTAATCACCATGCCGCGTGAAACCAGCAACCCGCTCGGCAGGGATCCCTCTTCTCGCACCGGGCCCATAGCTTGTGGGGGTAGCTAAATAATGATCTA | *Neophocaena asiaeorientalis* or *N. phocaenoides* | 99 | 99 | 1.21 × 10^4^ |
| B12 | 11-July-23 | 33°18'13.2"N | 126°47'21.9"E |  |  |  |  |  |
| B13 | 11-July-23 | 33°28'51.0"N | 126°54'14.1"E |  |  |  |  |  |
| B14 | 11-July-23 | 33°30'06.6"N | 126°54'49.6"E | CTTAATCACCATGCCGCGTGAAACCAGCAACCCGCTCGGCAGGGATCCCTCTTCTCGCACCGGGCCCATAGCTTGTGGGGGTAGCTAAATAATGATCT | *Neophocaena asiaeorientalis* or *N. phocaenoides* | 100 | 98.98 | 9.19 × 10^3^ |
| B15 | 11-July-23 | 33°32'04.2"N | 126°50'26.4"E |  |  |  |  |  |
| B16 | 11-July-23 | 33°33'38.1"N | 126°48'50.3"E | AGCTTAATCACCATGCCGCGTGAAACCAGCAACCCGCTCGGCAGGGATCCCTCTTCTCGCACCGGGCCCATAGCTTGTGGGGGTAGCTAAATAATGATCTA | *Neophocaena asiaeorientalis* or *N. phocaenoides* | 99 | 99 | 3.33 × 10^3^ |
| B17 | 11-July-23 | 33°33'29.3"N | 126°44'50.9"E | AGCTTAATCACCATGCCGCGTGAAACCAGCAACCCGCTCGGCAGGGATCCCTCTTCTCGCACCGGGCCCATAGCTTGTGGGGGTAGCTAAATAATGATCTA | *Neophocaena asiaeorientalis* or *N. phocaenoides* | 99 | 99 | 6.81 × 10^4^ |
| B18 | 12-July-23 | 33°33'04.5"N | 126°38'29.6"E |  |  |  |  |  |
| B19 | 12-July-23 | 33°31'40.6"N | 126°35'14.4"E |  |  |  |  |  |
| C01 | 07-Jun-22 | 33°58'54.7"N | 126°15'47.6"E | CGAGCTTAATCACCATGCCGCGTGAAACCAGCAACCCGCTCGGCAGGGATCCCTCTTCTCGCACCGGGCCCATAGCTTGTGGGGGTAGCTAAATAATGATCT | *Neophocaena asiaeorientalis* or *N. phocaenoides* | 100 | 99.02 | 6.12 × 10^3^ |
| C02 | 07-Jun-22 | 33°58'36.7"N | 126°19'36.7"E |  |  |  |  |  |
| C03 | 07-Jun-22 | 33°57'40.4"N | 126°21'37.1"E | TCACCATGCCGCGTGAAACCAGCAACCCGCTCGGCAAGGATCCCTCTTCTCGCACCGGGCCCATAGCTTGTGGGGGTAGCTAAATAATGATCTA | *Neophocaena asiaeorientalis* or *N. phocaenoides* | 98 | 97.85 | 1.36 × 10^4^ |
| C04 | 07-Jun-22 | 33°54'25.4"N | 126°18'43.9"E |  |  |  |  |  |
| C05 | 07-Jun-22 | 33°55'00.0"N | 126°22'08.2"E | TGCCGAGCGGGTTGCTGGTTTCACGCGGCATGGTGATTAAGCTCGTGATCTAATGGAGCGGCCATAGGATATAATGGTTATGAAGGGCTAGTGGACATAA | *Neophocaena asiaeorientalis* or *N. phocaenoides* | 99 | 100 | 1.38 × 10^4^ |
| C06 | 07-Jun-22 | 33°58'61.2"N | 126°19'61.1"E | CGCCGCGTGAAACCAGCAACCCGCTCGGCAGGGATCCCTCTTACTCGCACCGGGCCCATAGCTTGTGGGGGTAGCTAAATAATGATCTA | *Neophocaena asiaeorientalis* or *N. phocaenoides* | 97 | 97.7 | 1.36 × 10^4^ |
| C07 | 07-Jun-22 | 33°53'13.6"N | 126°29'39.4"E | CTTAATCACCATGCCGCGTGAAACCAGCAACCCGCTCGGCAGGGATCCCTCTTCTCGCACCGGGCCCATAGCTTGTGGGGGTAGCTAAATAATGATCTA | *Neophocaena asiaeorientalis* or *N. phocaenoides* | 98 | 98.98 | 7.59 × 10^3^ |
| C08 | 07-Jun-22 | 33°53'13.6"N | 126°29'39.4"E |  |  |  |  |  |
| C09 | 06-Jun-22 | 33°54'52.9"N | 126°39'43.2"E |  |  |  |  |  |
| C10 | 07-Jun-22 | 33°51'40.9"N | 126°18'02.6"E | GAGCTTAATCACCATGCCGCGTGAAACCAGCAACCCGCTCGGCAGGGATCCCTCTTCTCGCACCGGGCCCATAGCTTGTGGGGGTAGCTAAATAATGATCTA | *Neophocaena asiaeorientalis* or *N. phocaenoides* | 99 | 99.01 | 7.31 × 10^3^ |
| C11 | 07-Jun-22 | 33°47'43.8"N | 126°18'22.1"E | TCACCATCGCCGCGTGAAACCAGCAACCCGCTCGGCAAGGATCCCTCTTCTCGCACCGGGCCCATAGCTTGTGGGGGTAGCTAAATAATGATCTA | *Neophocaena asiaeorientalis* or *N. phocaenoides* | 98 | 96.81 | 6.65 × 10^3^ |
| C12 | 07-Jun-22 | 33°44'24.2"N | 126°21'23.0"E | TGCCGAGCGGGTTGCTGGTTTCACGCGGCATGGTGATTAAGCTCGTGATCTAATGGAGCGGCCATAGGATATAATGGTTATGAAGGGCTAGTGGACATA | *Neophocaena asiaeorientalis* or *N. phocaenoides* | 100 | 100 | 1.83 × 10^4^ |
| C13 | 07-Jun-22 | 33°40'15.7"N | 126°18'48.9"E | AGCTTAATCACCATGCCGCGTGAAACCAGCAACCCGCTCGGCAGGGATCCCTCTTCTCGCACCGGGCCCATAGCTTGTGGGGGTAGCTAAATAATGATCT | *Neophocaena asiaeorientalis* or *N. phocaenoides* | 100 | 99 | 7.63 × 10^3^ |
| D01 | 05-Jun-23 | 34°10'45.1"N | 126°11'38.1"E | CCATGCCGCGTGAAACCAGCAACCCGCTCGGCAGGGATCCCTCTTCTCGCACCGGGCCCATAGCTTGTGGGGGTAGCTAAATAATGATCT | *Neophocaena asiaeorientalis* or *N. phocaenoides* | 100 | 98.89 | 1.12 × 10^4^ |
| D02 | 03-Jun-23 | 34°02'48.0"N | 126°23'00.4"E |  |  |  |  |  |
| D03 | 02-Jun-23 | 34°01'05.8"N | 126°19'20.2"E |  |  |  |  |  |
| D04 | 02-Jun-23 | 33°59'03.1"N | 126°24'38.1"E | ATGCCGCGTGAAACCAGCAACCCGCTCGGCAGGGATCCCTCTTCTCGCACCGGGCCCATAGCTTGTGGGGGTAGCTAAATAATGATCTA | *Neophocaena asiaeorientalis* or *N. phocaenoides* | 98 | 98.86 | 6.44 × 10^3^ |
| D05 | 02-Jun-23 | 33°57'33.1"N | 126°21'26.6"E |  |  |  |  |  |
| D06 | 03-Jun-23 | 33°56'22.8"N | 126°21'12.9"E | TGCCGCGTGAAACCAGCAACCCGCTCGGCAGGGATCCCTCTTCTCGCACCGGGCCCATAGCTTGTGGGGGTAGCTAAATAAT | *Neophocaena asiaeorientalis* or *N. phocaenoides* | 100 | 98.78 | 9.07 × 10^3^ |
| D07 | 02-Jun-23 | 33°51'36.6"N | 126°20'09.9"E | CGGGTTGCTGGTTTCACGCGGCATGGTGATTAAGCTCGTGATCTAATGGAGCGGCCATAGGATATAATGGTTATAAAGGGC | *Neophocaena asiaeorientalis* or *N. phocaenoides* | 100 | 98.77 | 1.22 × 10^4^ |
| D08 | 02-Jun-23 | 33°53'45.4"N | 126°21'25.9"E | CCATGCCGCGTGAAACCAGCAACCCGCTCGGCAGGGATCCCTCTTCTCGCACCGGGCCCATAGCTTGTGGGGGTAGCTAAATAATGATCTA | *Neophocaena asiaeorientalis* or *N. phocaenoides* | 98 | 98.89 | 7.84 × 10^3^ |
| D09 | 02-Jun-23 | 33°47'16.2"N | 126°20'10.9"E | CCATGCCGCGTGAAACCAGCAACCCGCTCGGCAGGGATCCCTCTTCTCGCACCGGGCCCATAGCTTGTGGGGGTAGCTAAATAATGATCTC | *Neophocaena asiaeorientalis* or *N. phocaenoides* | 98 | 98.89 | 1.01 × 10^4^ |
| D10 | 02-Jun-23 | 33°43'20.3"N | 126°20'27.7"E | AGCTTAATCACCATGCCGCGTGAAACCAGCAACCCGCTCGGCAGGGATCCCTCTTCTCGCACCGGGCCCATAGCTTGTGGGGGTAGCTAAATAATGATCTA | *Neophocaena asiaeorientalis* or *N. phocaenoides* | 99 | 99 | 7.06 × 10^3^ |
| D11 | 02-Jun-23 | 33°39'51.2"N | 126°18'47.7"E |  |  |  |  |  |
| D12 | 02-Jun-23 | 33°32'03.7"N | 126°25'18.4"E |  |  |  |  |  |
| D13 | 02-Jun-23 | 33°32'03.7"N | 126°25'18.4"E |  |  |  |  |  |
| D14 | 06-Jun-23 | 33°57'59.3"N | 126°88'72.0"E |  |  |  |  |  |
| D15 | 06-Jun-23 | 33°43'55.5"N | 126°98'91.0"E | GCTCGGCAGGGATCCCTCTTCTCGCACCGGGCCCATAGCTTGTGGGGGTAGCTAAATAATGATCTA | *Neophocaena asiaeorientalis* or *N. phocaenoides* | 98 | 98.46 | 3.45 × 10^3^ |
| D16 | 06-Jun-23 | 33°35'06.3"N | 127°11'44.1"E |  |  |  |  |  |
| D17 | 02-Jun-23 | 33°16'49.3"N | 126°09'57.5"E |  |  |  |  |  |
| D18 | 01-Jun-23 | 33°10'36.0"N | 126°05'34.2"E |  |  |  |  |  |
| D19 | 01-Jun-23 | 33°07'14.7"N | 126°08'51.9"E |  |  |  |  |  |
| D20 | 01-Jun-23 | 33°08'04.6"N | 126°14'52.4"E | CACCATGCCGCGTGAAACCGCAACCCGCTCGGCAGGGATCCCTCTTCTCGCACCGGGCCCATAGCTTGTGGGGGTAGCTAAATAATGATCTA | *Neophocaena asiaeorientalis* or *N. phocaenoides* | 100 | 97.83 | 7.11 × 10^3^ |
| D21 | 01-Jun-23 | 33°08'15.4"N | 126°20'34.8"E | ATGCCGCGTGAAACCAGCAACCCGCTCGGCAGGGATCCCTCTTCTCGCACCGGGCCCATAGCTTGTGGGGGTAGCTAAATAATGATCTA | *Neophocaena asiaeorientalis* or *N. phocaenoides* | 98 | 98.86 | 1.02 × 10^4^ |
| D22 | 06-Jun-23 | 33°11'42.5"N | 126°31'07.5"E |  |  |  |  |  |
| D23 | 05-Jun-23 | 34°11'24.8"N | 127°07'42.8"E | CCATGCCGCGTGAAACCAGCAACCCGCTCGGCAGGGATCCCTCTTCTCGCACCGGGCCCATAGCTTGTGGGGGTAGCTAAATAATGATCT | *Neophocaena asiaeorientalis* or *N. phocaenoides* | 100 | 98.89 | 9.59 × 10^3^ |
| D24 | 05-Jun-23 | 34°16'57.5"N | 127°20'30.3"E |  |  |  |  |  |
| E01 | 11-May-22 | 36°45'00.0"N | 124°15'00.0"E | CTTAATCACCATGCCGCGTGAAACCAGCAACCCGCTCGGCAGGGATCCCTCTTCTCGCACCGGGCCCATAGCTTGTGGGGGTAGCTAAATAATGATCTATCTA | *Neophocaena asiaeorientalis* or *N. phocaenoides* | 95 | 98.98 | 1.22 × 10^4^ |
| E02 | 11-May-22 | 36°26'56.7"N | 123°27'08.3"E | TCACCATGCCGCGTGAAACCAGCAACCCGCTCGGCAGGGATCCCTCTTCTCGCACCGGGCCCATAGCTTGTGGGGGTAGCTAAATAATGATCTA | *Neophocaena asiaeorientalis* or *N. phocaenoides* | 98 | 98.92 | 1.14 × 10^4^ |
| E03 | 11-May-22 | 36°08'43.0"N | 123°27'46.6"E | CGAGCTTAATCACCATTGCCGCGTGAAACCAGCAACCCGCTCGGCAGGGATCCCTCTTCTCGCACCGGGCCCATAGCTTGTGGGGGTAGCTAAATAATGATCTA | *Neophocaena asiaeorientalis* or *N. phocaenoides* | 99 | 98.06 | 1.45 × 10^4^ |
| E04 | 11-May-22 | 36°07'13.9"N | 124°08'51.6"E | GAGCTTAATCACCATGCCGCGTGAAACCAGCAACCCGCTCGGCAGGGATCCCTCTTCTCGCACCGGGCCCATAGCTTGTGGGGGTAGCTAAATAATGATCTA | *Neophocaena asiaeorientalis* or *N. phocaenoides* | 99 | 99.01 | 1.73 × 10^4^ |
| E05 | 10-May-22 | 35°26'55.8"N | 124°07'43.3"E | CGAGCTTAATCACCATGCCGCGTGAAACCAGCAACCCGCTCGGCAGGGATCCCTCTTCTCGCACCGGGCCCATAGCTTGTGGGGGTAGCTAAATAATGATCTA | *Neophocaena asiaeorientalis* or *N. phocaenoides* | 99 | 99.02 | 1.44 × 10^4^ |
| E06 | 10-May-22 | 35°26'59.1"N | 123°26'51.7"E | CTTAATCACCATGCCGCGTGAAACCAGCAACCCGCTCGGCAGGGATCCCTCTTCTCGCACCGGGCCCATAGCTTGTGGGGGTAGCTAAATAATGATCTA | *Neophocaena asiaeorientalis* or *N. phocaenoides* | 98 | 98.98 | 1.04 × 10^4^ |
| E07 | 10-May-22 | 35°26'59.2"N | 123°08'34.6"E | ATCACGAGCTTAATCACCATGCCGCGTGAAACCAGCAACCCGCTCGGCAGGGATCCCTCTTCTCGCACCGGGCCCATAGCTTGTGGGGGTAGCTAAATAATGATCTAG | *Neophocaena asiaeorientalis* or *N. phocaenoides* | 98 | 99.06 | 1.14 × 10^4^ |
| E08 | 10-May-22 | 35°09'23.4"N | 122°27'18.8"E | GGAGCGGCCATAGGATATAATGGTTATGAAGGGCTAGTGGACATAA | *Neophocaena asiaeorientalis* or *N. phocaenoides* | 97 | 100 | 2.01 × 10^4^ |
| E09 | 09-May-22 | 34°26'44.7"N | 122°26'06.8"E |  |  |  |  |  |
| E10 | 09-May-22 | 34°09'38.3"N | 122°27'27.7"E |  |  |  |  |  |
| E11 | 09-May-22 | 34°09'02.5"N | 123°09'05.4"E |  |  |  |  |  |
| E12 | 09-May-22 | 34°08'40.9"N | 123°26'51.2"E |  |  |  |  |  |
| E13 | 08-May-22 | 33°27'03.6"N | 123°26'57.5"E |  |  |  |  |  |
| E14 | 08-May-22 | 33°26'19.4"N | 123°08'51.4"E |  |  |  |  |  |
| E15 | 08-May-22 | 33°08'25.2"N | 123°27'26.8"E | CACGCCGGCATGGTGATTAAGCTCCGTGATCTAATGGAGCGGCCATAGGATATAATGGTTATGAAGGGCTAGTGGACATAA | *Neophocaena asiaeorientalis* or *N. phocaenoides* | 98 | 97.5 | 9.76 × 10^3^ |
| E16 | 07-May-22 | 32°26'52.9"N | 124°08'09.4"E | GCTTCGTGATCTAATGGAGCGGCCATAGGATATAATGGTTATGAAGGGCTAGTGGACATAA | *Neophocaena asiaeorientalis* or *N. phocaenoides* | 93 | 100 | 9.61 × 10^3^ |
| E17 | 07-May-22 | 32°08'57.1"N | 124°26'46.6"E | AGCTTAATCACCATGCCGCGTGAAACCAGCAACCCGCTCGGCAGGGATCCCTCTTCTCGCACCGGGCCCATAGCTTGTGGGGGTAGCTAAATAAAAA | *Neophocaena asiaeorientalis* or *N. phocaenoides* | 96 | 98.94 | 1.2 × 10^4^ |
| E18 | 06-May-22 | 32°02'.89.9"N | 125°06'60.5"E | ACCATTCGCCGCGTGAAACCAGCAACCCGCTCGGCAGGGATCCCTCTTCTCGCACCGGGCCCATAGCTTGTGGGGGTAGCTAAATAATGATCTA | *Neophocaena asiaeorientalis* or *N. phocaenoides* | 98 | 96.77 | 7.35 × 10^3^ |
| F01 | 19-May-23 | 36°26'09.4"N | 123°32'16.0"E | AGCTTAATCACCATGCCGCGTGAAACCAGCAACCCGCTCGGCAGGGATCCCTCTTCTCGCACCGGGCCCATAGCTTGTGGGGGTAGCTAAA | *Neophocaena asiaeorientalis* or *N. phocaenoides* | 100 | 98.9 | 1.13 × 10^4^ |
| F02 | 19-May-23 | 36°13'10.2"N | 123°30'15.8"E | AGCTTAATCACCATGCCGCGTGAAACCAGCAACCCGCTCGGCAGGGATCCCTCTTCTCGCACCGGGCCCATAGCTTGTGGGGGTAGCTAAATAATGATCTA | *Neophocaena asiaeorientalis* or *N. phocaenoides* | 99 | 99 | 1.05 × 10^4^ |
| F03 | 19-May-23 | 36°08'32.5"N | 124°06'59.8"E | AGCTTAATCACCATGCCGCGTGAAACCAGCAACCCGCTCGGCAGGGATCCCTCTTCTCGCACCGGGCCCATAGCTTGTGGGGGTAGCTAAATAATGATCT | *Neophocaena asiaeorientalis* or *N. phocaenoides* | 100 | 99 | 1.17 × 10^4^ |
| F04 | 20-May-23 | 35°29'43.9"N | 124°06'39.6"E |  |  |  |  |  |
| F05 | 20-May-23 | 35°27'02.2"N | 123°27'04.0"E | AGCTTAATCACCATGCCGCGTGAAACCAGCAACCCGCTCGGCAGGGATCCCTCTTCTCGCACCGGGCCCATAGCTTGTGGGGGTAGCTAAATAATGATCTA | *Neophocaena asiaeorientalis* or *N. phocaenoides* | 99 | 99 | 1.14 × 10^4^ |
| F06 | 14-May-23 | 35°24'25.7"N | 123°08'33.3"E |  |  |  |  |  |
| F07 | 14-May-23 | 35°12'28.3"N | 122°34'16.5"E | CGCTCGGCAGGGATCCCTCTTCTCGCACCGGGCCCATAGCTTGTGGGGGTAGCTAAATAATGATCTA | *Neophocaena asiaeorientalis* or *N. phocaenoides* | 98 | 98.48 | 7.98 × 10^3^ |
| F08 | 14-May-23 | 35°10'02.7"N | 123°05'16.2"E | AGCTTAATCACCATGCCGCGTGAAACCAGCAACCCGCTCGGCAGGGATCCCTCTTCTCGCACCGGGCCCATAGCTTGTGGGGGTAGCTAAATAATGATCTA | *Neophocaena asiaeorientalis* or *N. phocaenoides* | 99 | 99 | 7.76 × 10^3^ |
| F09 | 13-May-23 | 35°06'52.6"N | 123°23'29.3"E |  |  |  |  |  |
| F10 | 13-May-23 | 34°29'03.2"N | 123°21'36.9"E |  |  |  |  |  |
| F11 | 13-May-23 | 34°26'51.2"N | 123°05'44.4"E | AGCTTAATCACCATGCCGCGTGAAACCAGCAACCCGCTCGGCAGGGATCCCTCTTCTCGCACCGGGCCCATAGCTTGTGGGGGTAGCTAAATAATGATCTA | *Neophocaena asiaeorientalis* or *N. phocaenoides* | 99 | 99 | 6.51 × 10^3^ |
| F12 | 13-May-23 | 34°23'54.7"N | 122°34'32.8"E | AGCTTAATCACCATGCCGCGTGAAACCAGCAACCCGCTCGGCAGGGATCCCTCTTCTCGCACCGGGCCCATAGCTTGTGGGGGTAGCTAAATAATGATCTA | *Neophocaena asiaeorientalis* or *N. phocaenoides* | 99 | 99 | 5.2 × 10^3^ |
| F13 | 12-May-23 | 34°08'53.2"N | 122°35'60.0"E | AGCTTAATCACCATGCCGCGTGAAACCAGCAACCCGCTCGGCAGGGATCCCTCTTCTCGCACCGGGCCCATAGCTTGTGGGGGTAGCTAAATAATGATCTA | *Neophocaena asiaeorientalis* or *N. phocaenoides* | 99 | 99 | 8.72 × 10^3^ |
| F14 | 12-May-23 | 34°09'02.2"N | 123°13'55.6"E | AGCTTAATCACCATGCCGCGTGAAACCAGCAACCCGCTCGGCAGGGATCCCTCTTCTCGCACCGGGCCCATAGCTTGTGGGGGTAGCTAAATAATGATCTA | *Neophocaena asiaeorientalis* or *N. phocaenoides* | 99 | 99 | 8.3 × 10^3^ |
| F15 | 12-May-23 | 34°06'55.1"N | 123°26'45.2"E |  |  |  |  |  |
| F16 | 12-May-23 | 33°27'07.1"N | 123°24'53.1"E |  |  |  |  |  |
| F17 | 11-May-23 | 33°18'43.7"N | 123°08'48.4"E |  |  |  |  |  |
| F18 | 11-May-23 | 33°08'16.1"N | 123°27'02.1"E |  |  |  |  |  |
| F19 | 11-May-23 | 32°26'59.8"N | 124°08'58.0"E | AGCTTAATCACCATGCCGCGTGAAACCAGCAACCCGCTCGGCAGGGATCCCTCTTCTCGCACCGGGCCCATAGCTTGTGGGGGTAGCTAAATAA | *Neophocaena asiaeorientalis* or *N. phocaenoides* | 100 | 98.94 | 6.94 × 10^3^ |
| F20 | 10-May-23 | 32°09'57.5"N | 124°29'02.9"E | TCACCATGCCGCGTGAAACCAGCAACCCGCTCGGCAGGGATCCCTCTTCTCGCACCGGGCCCATAGCTTGTGGGGGTAGCTAA | *Neophocaena asiaeorientalis* or *N. phocaenoides* | 100 | 98.8 | 7.02 × 10^3^ |
| G01 | 11-July-23 | 32°30'00.0"N | 126°00'00.0"E | AGCTTAATCACCATGCCGCGTGAAACCAGCAACCCGCTCGGCAGGGATCCCTCTTCTCGCACCGGGCCCATAGCTTGTGGGGGTAGCTAAATAATGATCTA | *Neophocaena asiaeorientalis* or *N. phocaenoides* | 99 | 99 | 8.98 × 10^3^ |
| G02 | 12-July-23 | 32°30'00.0"N | 127°00'00.0"E | CTCGGCAGGGATCCCTCTTCTCGCACCGGGCCCATAGCTTGTGGGGGTAGCTAAATAATGATCTA | *Neophocaena asiaeorientalis* or *N. phocaenoides* | 98 | 98.44 | 6.99 × 10^3^ |
| G03 | 12-July-23 | 32°00'00.0"N | 127°00'00.0"E | AGCTTAATCACCATGCCGCGTGAAACCAGCAACCCGCTCGGCAGGGATCCCTCTTCTCGCACCGGGCCCATAGCTTGTGGGGGTAGCTAAATAATGATCTA | *Neophocaena asiaeorientalis* or *N. phocaenoides* | 99 | 99 | 5.36 × 10^3^ |
| G04 | 12-July-23 | 32°00'00.0"N | 126°00'00.0"E | AGCTTAATCACCATGCCGCGTGAAACCAGCAACCCGCTCGGCAGGGACCCCTCTTCTCGCACCGGGCCCATAGCTTGTGGGGGTAGCTAAATAATGATCTA | *Neophocaena asiaeorientalis* or *N. phocaenoides* | 99 | 98 | 6.46 × 10^3^ |
| G05 | 12-July-23 | 31°30'00.0"N | 126°00'00.0"E |  |  |  |  |  |
| G06 | 12-July-23 | 31°30'00.0"N | 125°00'00.0"E |  |  |  |  |  |
