# Supplementary material for: Distribution analysis of the finless porpoises (Neophocaena sp.) and oceanic dolphins (Delphinidae) in the Korean Sea using environmental DNA
Source: PLoS One. 2025 May 16;20(5):e0322148. doi: 10.1371/journal.pone.0322148 (PMC12084060; doi:10.1371/journal.pone.0322148)
Supplement: S2 Table — Each sample was collected at the specified date along with its latitude and longitude. In cases where sequences were amplified from the samples using conventional PCR, sequencing and annotation were performed to investigate the most relevant organism. Query cover indicates the extent to which the target sequence encompasses the query sequence, expressed as a percentage, while percent identity represents the similarity of the matching portions between the target sequence and the query sequence, also expressed as a percentage. The concentration of eDNA was measured using qPCR. (DOCX) [file pone.0322148.s002.docx]

**S2 Table.** **The amplified eDNA results of the conventional PCR and qPCR examinations with primers Slo4-F & R.** Each sample was collected at the specified date along with its latitude and longitude. In cases where sequences were amplified from the samples using conventional PCR, sequencing and annotation were performed to investigate the most relevant organism. Query cover indicates the extent to which the target sequence encompasses the query sequence, expressed as a percentage, while percent identity represents the similarity of the matching portions between the target sequence and the query sequence, also expressed as a percentage. The concentration of eDNA was measured using qPCR.

| **No.** | **The date of sampling** | **Latitude** | **Longitude** | **Amplified sequence**  **(5’ – 3’)** | **Related organism** | **Query cover**  **(%)** | **Percentidentity**  **(%)** | **Concentration of eDNA (copies / µl)** |
| --- | --- | --- | --- | --- | --- | --- | --- | --- |
| A01 | 25-July-22 | 33°26'12.8"N | 126°16'17.8"E |  |  |  |  |  |
| A02 | 25-July-22 | 33°24'31.7"N | 126°15'13.3"E | GTGTGTGTGTGCATGCATGTGTGTGCATGTATGTGTGTGTGTGTGTGCAGGGGGTGATGTGGAATCTCCAAAGAGGGAGTTCTAATCACTGTGTGTCTTCTCATCAGAGTCTTTGTACCTGAAACTCAGGGAACATGGCTCCTCAGGTGAGGTAACCCCCGTGGGAATCACCACCTCCTCTCAA | *Tursiops aduncus* | 69 | 98.43 | 9.39 × 10^3^ |
| A03 | 25-July-22 | 33°39'13.0"N | 126°22'99.9"E |  |  |  |  |  |
| A04 | 25-July-22 | 33°21'44.3"N | 126°11'24.4"E |  |  |  |  |  |
| A05 | 25-July-22 | 33°21'13.7"N | 126°10'54.5"E | GTTCCCTGAGTTTCAGGTACAAAGACTCTGATGAGAAGACACACAGTGATTAGGACTCCCTCTTTGGAGATTCCACATCACCCCCTGCACACACGCACACACATACATGCACACACGTGCGTGCACACACACACGCGCGCGCGCGCGCGCACACACACCCTTTTTGTGTTTTCTC | *Delphinus delphis* | 78 | 97.2 | 1.25 × 10^4^ |
| A06 | 25-July-22 | 33°18'32.0"N | 126°09'52.9"E |  |  |  |  |  |
| A07 | 25-July-22 | 33°16'48.0"N | 126°10'10.2"E |  |  |  |  |  |
| A08 | 25-July-22 | 33°15'18.0"N | 126°11'32.6"E | TGTGTGTGTGTGCATGCATGTGTGTGCATGTATGTGTGTGTGTGTGTGCAGGGGGTGATGTGGAATCTCCAAAGAGGGAGTCCTAATCACTGTGTGTCTTCTCATCAGAGTCTTTGTACCTGAAACTCAGGGAACATGGCTCCTCAGGTGAGGTAACCCCCGTGGGAATCACCACCTCCTCTCACCT | *Orcinus orca* | 99 | 100 | 1.34 × 10^4^ |
| A09 | 26-July-22 | 33°14'35.2"N | 126°12'43.2"E |  |  |  |  |  |
| A10 | 26-July-22 | 33°14'17.5"N | 126°13'43.3"E | TGTGTGTGTGTGCATGCATGTGTGTGCATGTATGTGTGTGTGTGTGTGCAGGGGGTGATGTGGAATCTCCAAAGAGGGAGTCCTAATCACTGTGTGTCTTCTCATCAGAGTCTTTGTACCTGAAACTCAGGGAACATGGCTCCTCAGGTGAGGTAACCCCCGTGGGAATCACCACCTCCTCTCACC | *Orcinus orca* | 99 | 100 | 1.73 × 10^4^ |
| A11 | 26-July-22 | 33°13'53.8"N | 126°14'27.6"E |  |  |  |  |  |
| A12 | 26-July-22 | 33°13'29.6"N | 126°17'57.8"E |  |  |  |  |  |
| A13 | 26-July-22 | 33°14'26.5"N | 126°20'01.0"E |  |  |  |  |  |
| A14 | 26-July-22 | 33°14'39.5"N | 126°24'50.8"E | CGTGCGTGTGTGTGTGCATGCACGTGTGTGCATGTATGTGTGTGTGTGTGTGCAGGGGGTGATGTGGAATCTCCAAAGAGGGAGTCCTAATCACTGTGTGTCTTCTCATCAGAGTCTTTGTACCTGAAACTCAGGGAACATGGCTCCTCAGGTGAGGTAACCCCCGTGGGAATCACCACCTCCTCTCACCTTAA | *Orcinus orca* | 99 | 99.48 | 1.56 × 10^4^ |
| A15 | 26-July-22 | 33°14'17.5"N | 126°26'21.8"E | TGTGTGTGTGTGCATGCATGTGTGTGCATGTATGTGTGTGTGTGTGTGCAGGGGGTGATGTGGAATCTCCAAAGAGGGAGTCCTAATCACTGTGTGTCTTCTCATCAGAGTCTGTGTACCTGAAACTCAGGGAACATGGCTCCTCAGGTGAGGTAACCCCCGTGGGAATCACCACCTCCTCTCACCTTAA | *Orcinus orca* | 98 | 99.47 | 1.74 × 10^4^ |
| A16 | 26-July-22 | 33°13'36.8"N | 126°28'13.4"E |  |  |  |  |  |
| A17 | 27-July-22 | 33°14'40.2"N | 126°35'02.4"E |  |  |  |  |  |
| A18 | 27-July-22 | 33°15'02.9"N | 126°37'17.8"E | TGTGTGTGTGTGCATGCATGTGTGTGCATGTATGTGTGTGTGTGTGTGCAGGGGGTGATGTGGAATCTCCAAAGAGGGAGTCCTAATCACTGTGTGTCTTCTCATCAGAGTCTTTGTACCTGAAACTCAGGGAACATGGCTCCTCAGGTGAGGTAACCCCCGTGGGAATCACCACCTCCTCTCACCTTAA | *Orcinus orca* | 98 | 100 | 1.71 × 10^4^ |
| A19 | 27-July-22 | 33°16'08.0"N | 126°39'15.1"E |  |  |  |  |  |
| A20 | 27-July-22 | 33°16'11.3"N | 126°41'30.1"E | TGTTCCCTGAGTTTCAGGTACACAGACTCTGATGAGAAGACACACAGTGATTAGGACTCCCTCTTTGGAGATTCCACATCACCCCCTGCACACACACACACATATATGTGCACACGTGTGTGTGCACACACACACACGCACGCACGCACACACACACCCTTCCTGTAT | *Delphinus delphis* | 72 | 96.38 | 1.42 × 10^4^ |
| A21 | 27-July-22 | 33°17'15.0"N | 126°45'14.8"E | TGTTTCCTGAGTTTCAGGTACAAAGACTCTGATGAGAAGACACACAGTGATTAGGACTCCCTCTTTGGAGATTCCACATCACCCCCCGTACACACACACACACATACATGCACACACATGCATGCACACACACACACGCACGCACGCACGCACACACACACCCTTCCTGTATTCTCTCAACCGTGA | *Delphinus delphis* | 80 | 97.32 |  |
| A22 | 27-July-22 | 33°18'13.7"N | 126°47'22.2"E |  |  |  |  |  |
| A23 | 27-July-22 | 33°19'21.0"N | 126°50'48.8"E |  |  |  |  |  |
| A24 | 27-July-22 | 33°20'57.1"N | 126°51'48.6"E |  |  |  |  |  |
| A25 | 28-July-22 | 33°24'18.0"N | 126°54'18.7"E |  |  |  |  |  |
| A26 | 28-July-22 | 33°25'59.5"N | 126°55'45.1"E | GACTCCCTCTTTGGAGATTCCACATCACCCCCTGCACACACACACACACATACATGCACACACATGCATGCACACACACACACGCACGCACGCACGCACACACACACCCTTCCTGTATTCTCTCAAGCGTGA | *Delphinus delphis* | 72 | 98.95 | 1.79 × 10^4^ |
|  |  |  |  | GTGTGTGTGCATGCATGTGTGTGCATGTATGTGTGTGTGTGTGTGCAGGGGGTGATGTGGAATCTCCAAAGAGGGAGTCCTAATCACTGTGTGTCTTCTCATCAGAGTCTTTGTACCTGAAACTCAGGGAACATGGCTCCTCAGGTGAGGTAACCCCCGTGGGAATCACCACCTCCTCTCACCTTAA | *Tursiops aduncus* | 67 | 99.2 |  |
| A27 | 28-July-22 | 33°30'45.0"N | 126°53'54.6"E |  |  |  |  |  |
| A28 | 28-July-22 | 33°32'01.0"N | 126°50'24.4"E |  |  |  |  |  |
| A29 | 28-July-22 | 33°33'35.6"N | 126°48'49.7"E |  |  |  |  |  |
| A30 | 28-July-22 | 33°33'29.5"N | 126°44'55.0"E |  |  |  |  |  |
| A31 | 28-July-22 | 33°32'55.7"N | 126°41'02.8"E |  |  |  |  |  |
| A32 | 28-July-22 | 33°33'11.2"N | 126°38'37.0"E | GATTAGGACTCCCTCTTTGGAGATTCCACATCACCCCCTGCACACACACACACATACATGCACACACATGCATGCACACACACACACGCACGCACGCACGCACACACACACCCTTCCTGTATTCTCTCAACC | *Delphinus delphis* | 75 | 97.03 | 1.22 × 10^4^ |
|  |  |  |  |  | *Stenella longirostris* | 100 | 99.24 |  |
| A33 | 29-July-22 | 33°31'32.5"N | 126°35'01.3"E | TGAGTTTCAGGTACAAAGACTCTGATGAGAAGACACACAGTGATTAGGACTCCCTCTTTGGAGATTCCACATCACCCCCTGCACACACACACACACATACATGCACACACATGCATGCACACACACACACGCACGCACGCACGCACACACATACCCTTCCTGTATTCTCTCAACCG | *Tursiops aduncus* | 94 | 99.4 | 1.53 × 10^4^ |
| A34 | 29-July-22 | 33°31'36.8"N | 126°33'50.0"E | TTTCAGGTACAAAGACTCTGATGAGAAGACACACAGTGATTAGGACTCCCTCTTTGGAGATTCCACATCACCCCCTGCACACACACACACACATACATGCACACACATGCATGCACACACACACACGCACGCACGCACGCACACACATACCCTTCCTGTATTCTCTCAACCG | *Tursiops aduncus* | 95 | 99.39 | 1.73 × 10^4^ |
| A35 | 29-July-22 | 33°31'36.8"N | 126°33'50.0"E |  |  |  |  |  |
| A36 | 29-July-22 | 33°29'53.9"N | 126°27'11.5"E | TGAGTTTCAGGTACAAAGACTCTGATGAGAAGACACACAGTGATTAGGACTCCCTCTTTGGAGATTCCACATCACCCCCTGCACACACACACACACATACATGCACACACATGCATGCACACACACACACGCACGCACGCACGCACACACATACCCTTCCTGTATTCTCTCAACCGTGA | *Tursiops aduncus* | 93 | 99.4 | 1.28 × 10^4^ |
| A37 | 29-July-22 | 33°29'02.8"N | 126°23'42.0"E | GACTCCCTCTTTGGAGATTCCACATCACCCCCTGCACACACACACACACATACATGCACACACATGCATGCACACACACACACGCACGCACGCACGCACACACACACCCTTCCTGTATTCTCTCAACCGTGA | *Delphinus delphis* | 72 | 98.95 | 1.41 × 10^4^ |
| A38 | 29-July-22 | 33°28'21.4"N | 126°20'54.2"E |  |  |  |  |  |
| A39 | 29-July-22 | 33°27'12.2"N | 126°18'25.2"E | TGAGTTTCAGGTACAAAGACTCTGATGAGAAGACACACAGTGATTAGGACTCCCTCTTTGGAGATTCCACATCACCCCCTGCACACACACACACGCATACATGCACACACATGCATGCACACACACACACGCACGCACGCACGCACACACATACCCTTCCTGTATTCTCTCAA | *Tursiops aduncus* | 96 | 98.8 | 2.16 × 10^4^ |
| B01 | 10-July-23 | 33°29'39.5"N | 126°25'57.5"E |  |  |  |  |  |
| B02 | 10-July-23 | 33°28'24.4"N | 126°20'59.5"E |  |  |  |  |  |
| B03 | 10-July-23 | 33°26'13.1"N | 126°16'18.0"E | GTTCCCTGAGTTTCAGGTACAAAGACTCTGATGAGAAGACACACGGTGATTAGGACTCCCTCTTTGGAGATTCCACATCACCCCCTGCACACACACACATACATGCACGCACATGCATGCACGCACACAC | *Tursiops aduncus* | 95 | 93.7 | 2.02 × 10^4^ |
| B04 | 10-July-23 | 33°24'10.0"N | 126°15'02.6"E |  |  |  |  |  |
| B05 | 10-July-23 | 33°21'44.0"N | 126°11'23.8"E |  |  |  |  |  |
| B06 | 10-July-23 | 33°16'42.0"N | 126°10'09.7"E |  |  |  |  |  |
| B07 | 10-July-23 | 33°15'52.1"N | 126°10'50.4"E |  |  |  |  |  |
| B08 | 10-July-23 | 33°14'32.5"N | 126°12'44.0"E | TGTTCCCTGAGTTTCAGGTACAAAGACTCTGATGAGAAGACACACAGTGATTAGGACTCCCTCTTTGGAGATTCCACATCACCCCCTGCACACACACACACACATACATGCACACACATGCATGCACACACACACACGCACGCACGCACGCACACACACACCCTT | *Orcinus orca* | 100 | 98.79 | 1.25 × 10^4^ |
|  |  |  |  | CGTGTGTGTGTGTGCATGCATGTGTGTGCATGTATGTGTGTGTGTGTGTGCAGGGGGAGATGTGAAATCTCCAAAGAGGGAGTCCTAATCACTGTGTGTCCTCTCATCAGACTCTTTGTACCTGAAACTCAGGAAACATGGCTCCTCAGGAGAGGTAACCCCCGTGAGAATCACCCCCTCCTCTCACCAAAA | *Delphinus delphis* | 72 | 96.38 |  |
| B09 | 10-July-23 | 33°14'17.7"N | 126°13'42.5"E |  |  |  |  |  |
| B10 | 11-July-23 | 33°14'39.4"N | 126°24'50.3"E |  |  |  |  |  |
| B11 | 11-July-23 | 33°16'10.7"N | 126°40'23.5"E | ATAACCTCACCTGTGTTCTTTGCTCTCTAACCCCTCTTGGTTCCAGCTGATGTTTCATCTTAGCTCTCAGTCTTCCTGTATTCTCTCAACCGTGGTCTTCCTGTATTCTCTCAACCGTG | *Delphinus delphis* | 72 | 94.19 | 7.21 × 10^3^ |
| B12 | 11-July-23 | 33°18'13.2"N | 126°47'21.9"E |  |  |  |  |  |
| B13 | 11-July-23 | 33°28'51.0"N | 126°54'14.1"E |  |  |  |  |  |
| B14 | 11-July-23 | 33°30'06.6"N | 126°54'49.6"E |  |  |  |  |  |
| B15 | 11-July-23 | 33°32'04.2"N | 126°50'26.4"E |  |  |  |  |  |
| B16 | 11-July-23 | 33°33'38.1"N | 126°48'50.3"E |  |  |  |  |  |
| B17 | 11-July-23 | 33°33'29.3"N | 126°44'50.9"E |  |  |  |  |  |
| B18 | 12-July-23 | 33°33'04.5"N | 126°38'29.6"E |  |  |  |  |  |
| B19 | 12-July-23 | 33°31'40.6"N | 126°35'14.4"E |  |  |  |  |  |
| C01 | 07-Jun-22 | 33°58'54.7"N | 126°15'47.6"E |  |  |  |  |  |
| C02 | 07-Jun-22 | 33°58'36.7"N | 126°19'36.7"E |  |  |  |  |  |
| C03 | 07-Jun-22 | 33°57'40.4"N | 126°21'37.1"E | TGCGTGCGTGTGTGTGTGTGCATGCATGTGTGTGCATGTATGTGTGTGTGTGTGCAGGGGGTGATGTGGAATCTCCAAAGAGGGAGTCCTAATCACTGTGTGTCTTCTCATCAGAGTCTTTGTACCTGAAACTCAGGGAACATGGCTCCTCAGGTGAGGTAACCCCCGTGGGAATCACCACCTCCTCTCACCTTAA | *Orcinus orca* | 99 | 98.4 | **1.01 × 10^5^** |
|  |  |  |  | CCATGTTCCCTGAGTTTCAGGTACAAAGACTCTGATGAGAAGACACACAGTGATTAGGACTCCCTCTTTGGAGATTCCACATCACCCCCTGCACACACACACACACATACATGCACACACATGCATGCACACACACACACGCACGCACGCACGCACACACATACCCTTCCTGTATTCTCTCAACCGTGACCC | *Tursiops aduncus* | 86 | 99.4 |  |
| C04 | 07-Jun-22 | 33°54'25.4"N | 126°18'43.9"E | TGCGTGCGTGTGTGTGTGTGCATGCATGTGTGTGCATGTATGTGTGTGTGTGTGTGCAGGGGGTGATGTGGAATCTCCAAAGAGGGAGTCCTAATCACTGTGTGTCTTCTCATCAGAGTCTTTGTACCTGAAACTCAGGGAACATGGCTCCTCAGGTGAGGTAACCCCCGTGGGAATCACCACCTCCTCTCACCTTAA | *Orcinus orca* | 99 | 98.99 | **1.64 × 10^5^** |
| C05 | 07-Jun-22 | 33°55'00.0"N | 126°22'08.2"E |  |  |  |  |  |
| C06 | 07-Jun-22 | 33°58'61.2"N | 126°19'61.1"E |  |  |  |  |  |
| C07 | 07-Jun-22 | 33°53'13.6"N | 126°29'39.4"E |  |  |  |  |  |
| C08 | 07-Jun-22 | 33°53'13.6"N | 126°29'39.4"E | TGAGTTTCAGGTACAAAGACTCTGATGAGAAGACACACAGTGATTAGGACTCCCTCTTTGGAGATTCCACATCACCCCCTGCACACACACACACACATACATGCACACACATGCGTGCACACACACACACGCACGCACGCGCGCACACACATACCCTT | *Tursiops aduncus* | 99 | 93.71 | **3.66 × 10^5^** |
| C09 | 06-Jun-22 | 33°54'52.9"N | 126°39'43.2"E | TGAGTTTCAGGTACAAAGACTCTGATGAGAAGACACACAGTGATTAGGACTCCCTCTTTGGAGATTCCACATCACCCCCTGCACACACACACACATACATGCACACACATGCGTGCACACACACACGCACGCACGCACGCACACACACACCCTTCCTGTATTCTCTC | *Tursiops aduncus* | 98 | 95.81 | 9.26 × 10^4^ |
|  |  |  |  | TGAGTTTCAGGTACAAAGACTCTAGATAGAGAAGACACACAGTGATTAGGACTCCCTCTTTGGAGATTCCACATCACCCCCTGCACACACACACACATATACATGCACACACATGCATGCACACACACACACACACACACACACACACACACACCCTT | *Tursiops aduncus* | 99 | 93.71 |  |
| C10 | 07-Jun-22 | 33°51'40.9"N | 126°18'02.6"E |  |  |  |  |  |
| C11 | 07-Jun-22 | 33°47'43.8"N | 126°18'22.1"E | TGCGTGCGTGTGTGTGTGTGCATGCATGTGTGTGCATGTATGTGTGTGCGTGTGTGCAGGGGGTGATGTGGAATCTCCAAAGAGGGAGTCCTAATCACTGTGTGTCTTCTCATCAGAGTCTTTGTACCTGAAACTCAGGGAACATGGCTCCTCAGGTGAGGTAACCCCCGTGGGAATCACCACCTCCTCTCACCTTAA | *Orcinus orca* | 99 | 98.49 | 4.76 × 10^4^ |
| C12 | 07-Jun-22 | 33°44'24.2"N | 126°21'23.0"E | TGCGTGCGTGTGTGTGTGTGCATGCATGTGTGTGCATGTATGTGTGTGTGTGTGTGCAGGGGGTGATGTGGAATCTCCAAAGAGGGAGTCCTAATCACTGTGTGTCTTCTCATCAGAGTCTTTGTACCTGAAACTCAGGGAACATGGCTCCTCAGGTGAGGTAACCCCCGTGGGAATCACCACCTCCTCTCACCT | *Orcinus orca* | 100 | 98.98 | **1.23 × 10^5^** |
|  |  |  |  | TGCGTGTGTGTGTGTGCATGCATGTGTGTGCATGTATGTGTGTGTGTGTGTGCAGGGGGTGATGTGGAATCTCCAAAGAGGGAGTCCTAATCACTGTGTGTCTTCTCATCAGAGTCTTTGTACCTGAAACTCAGGGAACATGGCTCCTCAGGTGAGGTAACCCCCGTGGGAATCACCACCTCCTCTCACCTTAA | *Orcinus orca* | 99 | 98.99 |  |
| C13 | 07-Jun-22 | 33°40'15.7"N | 126°18'48.9"E |  |  |  |  |  |
| D01 | 05-Jun-23 | 34°10'45.1"N | 126°11'38.1"E |  |  |  |  |  |
| D02 | 03-Jun-23 | 34°02'48.0"N | 126°23'00.4"E |  |  |  |  |  |
| D03 | 02-Jun-23 | 34°01'05.8"N | 126°19'20.2"E | GTACAAAGACTCTGATGAGAAGACACACAGTGATTAGGACTCCCTCTTTGGAGATTCCACATCACCCCCTGCACACACACACACACATACATGCACACACGTGCATGCACACACACA | *Tursiops aduncus* | 100 | 99.15 | 1.44 × 10^4^ |
| D04 | 02-Jun-23 | 33°59'03.1"N | 126°24'38.1"E |  |  |  |  |  |
| D05 | 02-Jun-23 | 33°57'33.1"N | 126°21'26.6"E | GTACAAAGACTCTGATGAGAAGACACACAGTGATTAGGACTCCCTCTTTGGAGATTCCACATCACCCCCTGCACACACACACACACATACATGCACACACATGCATGCACACACACAC | *Tursiops aduncus* | 100 | 100 | 1.41 × 10^4^ |
| D06 | 03-Jun-23 | 33°56'22.8"N | 126°21'12.9"E |  |  |  |  |  |
| D07 | 02-Jun-23 | 33°51'36.6"N | 126°20'09.9"E |  |  |  |  |  |
| D08 | 02-Jun-23 | 33°53'45.4"N | 126°21'25.9"E |  |  |  |  |  |
| D09 | 02-Jun-23 | 33°47'16.2"N | 126°20'10.9"E |  |  |  |  |  |
| D10 | 02-Jun-23 | 33°43'20.3"N | 126°20'27.7"E | CGTGCGTGTGTGTGTGTGCATGCATGTGTGTGCATGTATGTGTGTGTGTGTGTGCAGGGGGTGATGTGGAATCTCCAAAGAGGGAGTCCTAATCACTGTGTGTCTTCTCATCAGAGTCTTTGTACCTGAAACTCAGGGAACATGGCTCCTCAGGTGAGGTAACCCCCGTGGGAATCACCACCTCCTCTCACC | *Orcinus orca* | 100 | 98.97 | 2.17 × 10^4^ |
| D11 | 02-Jun-23 | 33°39'51.2"N | 126°18'47.7"E |  |  |  |  |  |
| D12 | 02-Jun-23 | 33°32'03.7"N | 126°25'18.4"E |  |  |  |  |  |
| D13 | 02-Jun-23 | 33°32'03.7"N | 126°25'18.4"E | TGTGTGTGTGTGCATGCATGTGTGTGCATGTATGTGTGTGTGTGTGTGCACGGGGTGATGTGGAATCTCCAAAGAGGGAGTCCTAATCTCTGTGTGTCTTCTCATCAGAGTCTTTGTACCTGAAACTCAGGGAACATGGCTCCTCAGGTGAGGTAACCCCCGTGGGAATCACCCCCTCCTCTCACCTTAAAA | *Orcinus orca* | 97 | 98.4 | 1.56 × 10^4^ |
| D14 | 06-Jun-23 | 33°57'59.3"N | 126°88'72.0"E |  |  |  |  |  |
| D15 | 06-Jun-23 | 33°43'55.5"N | 126°98'91.0"E | GACTCCCTCTTTGGAGATTCCACATCACCCCCTGCACACACACACACACATACATGCACACACATGCATGCACACACACACACGCACGCACGCACGCACACACATAC | *Delphinus delphis* | 89 | 98.95 | 1.63 × 10^4^ |
| D16 | 06-Jun-23 | 33°35'06.3"N | 127°11'44.1"E |  |  |  |  |  |
| D17 | 02-Jun-23 | 33°16'49.3"N | 126°09'57.5"E | TGTTCCCTGAGTTTCAGGTACAAAGACTCTGATGAGAAGACACACAGTGATTAGGACTCCCTCTTTGGAGATTCCACATCACCCCCTGCACACACACACACACATACATGCACACACATGCATGCACACACACACACGCACGCACGCACGCACACACATACCCTTCCTGTATTCTCTCAACCGTGA | *Delphinus delphis* | 80 | 99.33 | 1.92 × 10^4^ |
| D18 | 01-Jun-23 | 33°10'36.0"N | 126°05'34.2"E | TGCGTGTGTGTGTGTGCATGCATGTGTGTGCATGTATGTGTGTGTGTGTGTGCAGGGGGTGATGTGGAATCTCCAAAGAGGGAGTCCTAATCACTGTGTGTCTTCTCATCAGAGTCTTTGTACCTGAAACTCAGGGAACATGGCTCCTCAGGTGAGGTAACCCCCGTGGGAATCACCACCTCCTCTCACCTTAA | *Orcinus orca* | 99 | 98.97 | 1.38 × 10^4^ |
| D19 | 01-Jun-23 | 33°07'14.7"N | 126°08'51.9"E |  |  |  |  |  |
| D20 | 01-Jun-23 | 33°08'04.6"N | 126°14'52.4"E | GGTACAAAGACTCTGATGAGAAACACACAGTGATTAGGACTCCCTCTTTGGAGATTCCACATCACCCCCTGCACACACACACACACATACATGCACACACATGCGTGCACACACACACACGCACGCACGCACGCACACACATACCCTTCCTGTATTCTCTCAA | *Tursiops aduncus* | 96 | 98.74 | 1.59 × 10^4^ |
| D21 | 01-Jun-23 | 33°08'15.4"N | 126°20'34.8"E |  |  |  |  |  |
| D22 | 06-Jun-23 | 33°11'42.5"N | 126°31'07.5"E |  |  |  |  |  |
| D23 | 05-Jun-23 | 34°11'24.8"N | 127°07'42.8"E |  |  |  |  |  |
| D24 | 05-Jun-23 | 34°16'57.5"N | 127°20'30.3"E |  |  |  |  |  |
| E01 | 11-May-22 | 36°45'00.0"N | 124°15'00.0"E | GACTCCCTCTTTGGAGATTCCACATCACCCCCTGCACACACACACACACATACATGCACACACATGCATGCACACACACACACGCACGCACGCACGCACACACACACCCTTCCTGTATTCTCTCAA | *Delphinus delphis* | 75 | 98.95 | 1.86 × 10^4^ |
| E02 | 11-May-22 | 36°26'56.7"N | 123°27'08.3"E | GGTGGGCATGGCCAGAAGGACAGGGGTGAGGGGAGAAGGAAAAAGAGCAACAGGAAGATCTACAAGAAGCCCGACCCCTCCCAGGGCCTCCCCCCATCACCACCTCCTCTCACCTTAA | *Delphinus delphis* | 96 | 97.39 | 2.24 × 10^4^ |
| E03 | 11-May-22 | 36°08'43.0"N | 123°27'46.6"E | ATCTTCCTGTTGCTCTTTTTCCTTCTCCCCTCACCCCTGTCCTTCTGGCCATGCCCACCTTGGTCTTGTTATCTCTCTCTATCTCTTTGTGTCTTCCTGTATTCTCTCAACCGTG | *Delphinus delphis* | 93 | 96.33 | 1.9 × 10^4^ |
| E04 | 11-May-22 | 36°07'13.9"N | 124°08'51.6"E | TCGCTGCAGAAGTGGGGGTGGGAAGGCTGTAGGGCTCCTCCGACAGCACAGTCTCTGTGGCTCCCTAAGTGGGCCGTTCCTGTATTCTCT | *Delphinus delphis* | 95 | 97.67 | 1.39 × 10^4^ |
| E05 | 10-May-22 | 35°26'55.8"N | 124°07'43.3"E |  |  |  |  |  |
| E06 | 10-May-22 | 35°26'59.1"N | 123°26'51.7"E |  |  |  |  |  |
| E07 | 10-May-22 | 35°26'59.2"N | 123°08'34.6"E | TGCGTGTGTGTGTGTGCATGCATGTGTGTGCATGTATGTGTGTGTGTGTGTGCAGGGGGTGATGTGGAATCTCCAAAGAGGGAGTCCTAATCACTGTGTGTCTTCTCATCAGAGTCTTTGTACCTGAAACTCAGGGAACATGGCTCCTCAGGTGAGGTAACCCCCGTGGGAATCACCACCTCCTCTC | *Orcinus orca* | 100 | 98.94 | 4.09 × 10^4^ |
| E08 | 10-May-22 | 35°09'23.4"N | 122°27'18.8"E | TGTGTGTGTGCATGCATGTGTGTGCATGTATGTGTGTGTGTGTGTGCAGGGGGTGATGTGGAATCTCCAAAGAGGGAGTCCTAATCACTGTGTGTCTTCTCATCAGAGTCTTTGTACCTGAAACTCAGGAAACATGGCTCCTCAGGTGAGGTAACCCCCGTGGGAATCACCACCTCCTCTCACCTTAA | *Orcinus orca* | 99 | 99.47 | 2.97 × 10^4^ |
| E09 | 09-May-22 | 34°26'44.7"N | 122°26'06.8"E |  |  |  |  |  |
| E10 | 09-May-22 | 34°09'38.3"N | 122°27'27.7"E |  |  |  |  |  |
| E11 | 09-May-22 | 34°09'02.5"N | 123°09'05.4"E |  |  |  |  |  |
| E12 | 09-May-22 | 34°08'40.9"N | 123°26'51.2"E | CGTGTGTGTGTGTGCATGCATGTGTGTGCATGTATGTGTGTGTGTGTGTGCAGGGGGTGATGTGGAATCTCCAAAGAGGGAGTCCTAATCACTGTGTGTCTTCTCATCAGAGTCTTTGTACCTGAAACTCAGGGAACATGGCTCCTCAGGTGAGGTAACCCCCGTGGG | *Orcinus orca* | 98 | 100 | **1.38 × 10^6^** |
| E13 | 08-May-22 | 33°27'03.6"N | 123°26'57.5"E | CGTGTGTGTGTGTGCATGCATGTGTGTGCATGTATGTGTGTGTGTGTGTGCAGGGGGTGATGTGGAATCTCCAAAGAGGGAGTCCTAATCACTGTGTGTCTTCTCATCAGAGTCTTTGTACCTGAAACTCAGGGAACATGGCTCCTCAGGTGAGGTAACCCCCGTGGGAATCACCACCTCCTCTCACC | *Orcinus orca* | 98 | 100 | 2.34 × 10^4^ |
| E14 | 08-May-22 | 33°26'19.4"N | 123°08'51.4"E |  |  |  |  |  |
| E15 | 08-May-22 | 33°08'25.2"N | 123°27'26.8"E |  |  |  |  |  |
| E16 | 07-May-22 | 32°26'52.9"N | 124°08'09.4"E |  |  |  |  |  |
| E17 | 07-May-22 | 32°08'57.1"N | 124°26'46.6"E |  |  |  |  |  |
| E18 | 06-May-22 | 32°02'.89.9"N | 125°06'60.5"E | GACTCCCTCTTTGGAGATTCCACATCACCCCCTGCACACACACACACACATACATGCACACACATGCATGCACACACACACACGCACGCACGCACGCACACACATACCCTTCCTGTATTCTCTCAA | *Delphinus delphis* | 75 | 98.95 | 1.63 × 10^4^ |
| F01 | 19-May-23 | 36°26'09.4"N | 123°32'16.0"E |  |  |  |  |  |
| F02 | 19-May-23 | 36°13'10.2"N | 123°30'15.8"E | TGTTCCCTGAGTTTCAGGTACAAAGACTCTGATGAGAAGACACACAGTGATTAGGACTCCCTCTTTGGAGATTCCACATCACCCCCTGCACACACACACATACATGCACACACATGCATGCACACACACACACGCACGCACGCACGCACACACATACCCTTCCTGTATTCTCTCAACCGTGAGAACATGTTTTGTG | *Delphinus delphis* | 74 | 96.64 | **1.96 × 10^6^** |
| F03 | 19-May-23 | 36°08'32.5"N | 124°06'59.8"E | TGTGTGTGTGCGCATGCATGTGTGTGCATGTATGTGTGTGTGTGCAGGGGGTGATGTGGAATCTCCAAAGAGGGAGTCCTAATCACTGTGTGTCTTCTCATCAGAGTCTTTGTACCTGAAACTCAGGGAACATGGCTCCTCAGGTGAGGTAACCCCCGTGGGAATCACCACCTCCTCTCACCTTAA | *Orcinus orca* | 98 | 97.34 | 7.85 × 10^4^ |
| F04 | 20-May-23 | 35°29'43.9"N | 124°06'39.6"E | TGTGTGTGTGTGCATGCATGTGTGTGCATGTATGTGTGTGTGTGTGTGCAGGGGGTGATGTGGAATCTCCAAAGAGGGAGTCCTAATCACTGTGTGTCTTCTCATCAGAGTCTTTGTACCTGAAACTCAGGGAACATGGCTCCTCAGGTGAGGTAACCCCCGTGGGAATCACCACCTCCTCTCACCTTAA | *Orcinus orca* | 98 | 100 | 1.15 × 10^4^ |
| F05 | 20-May-23 | 35°27'02.2"N | 123°27'04.0"E | TGTTCCCTGAGTTTCAGGTACAAAGACTCTGATGAGAAGACACACAGTGATTAGGACTCCCTCTTTGGAGATTCCACATCACCCCCTGCACACACACACACACATACATGCACACACATGCATGCACACACACACACGCACGCACGCACGCACACACATACCCTTCCTGTATTCTCTCAACCGTGA | *Delphinus delphis* | 80 | 99.33 | **2.25 × 10^5^** |
| F06 | 14-May-23 | 35°24'25.7"N | 123°08'33.3"E |  |  |  |  |  |
| F07 | 14-May-23 | 35°12'28.3"N | 122°34'16.5"E |  |  |  |  |  |
| F08 | 14-May-23 | 35°10'02.7"N | 123°05'16.2"E |  |  |  |  |  |
| F09 | 13-May-23 | 35°06'52.6"N | 123°23'29.3"E |  |  |  |  |  |
| F10 | 13-May-23 | 34°29'03.2"N | 123°21'36.9"E |  |  |  |  |  |
| F11 | 13-May-23 | 34°26'51.2"N | 123°05'44.4"E |  |  |  |  |  |
| F12 | 13-May-23 | 34°23'54.7"N | 122°34'32.8"E | TGTTCCCTGAGTTTCAGGTACAAAGACTCTGATGAGAAGACACACAGTGATTAGGACTCCCTCTTTGGAGATTCCACATCACCCCCTGCACACACACACACACATACATGCACACACATGCATGCACACACACACACGCACGCACGCACGCACACACATACCCTTCCTGTATTCTCTCAACCGTGA | *Delphinus delphis* | 80 | 99.33 | 5.11 × 10^4^ |
| F13 | 12-May-23 | 34°08'53.2"N | 122°35'60.0"E |  |  |  |  |  |
| F14 | 12-May-23 | 34°09'02.2"N | 123°13'55.6"E |  |  |  |  |  |
| F15 | 12-May-23 | 34°06'55.1"N | 123°26'45.2"E | TGTGTGTGTGCGCATGCATGTGTGTGCATGTATGTGTGTGTGTGTGTGCAGGGGGTGATGTGGAATCTCCAAAGAGGGAGTCCTAATCACTGTGTGTCTTCTCATCAGAGTCTTTGTACCTGAAACTCAGGGAACATGGCTCCTCAGGTGAGGTAACCCCCGTGGGAATCACCACCTCCTCTCACCTTAA | *Orcinus orca* | 98 | 99.47 | 2.66 × 10^4^ |
| F16 | 12-May-23 | 33°27'07.1"N | 123°24'53.1"E |  |  |  |  |  |
| F17 | 11-May-23 | 33°18'43.7"N | 123°08'48.4"E |  |  |  |  |  |
| F18 | 11-May-23 | 33°08'16.1"N | 123°27'02.1"E | TGCGTGTGTGTGTGTGCATGCATGTGTGTGCATGTATGTGTGTGTGTGTGTGCAGGGGGTGATGTGGAATCTCCAAAGAGGGAGTTCTAATCACTGTGTGTCTTCTCATCAGAGTCTTTGTACCTGAAACTCAGGGAACATGGCTCCTCAGGTGAGGTAACCCCCGTGGGAATCACCACCTCCTCTCA | *Orcinus orca* | 100 | 98.42 | 3.49 × 10^4^ |
| F19 | 11-May-23 | 32°26'59.8"N | 124°08'58.0"E | TGCGTGCGTGTGTGTGTGCGCATGCATGTGTGTGCATGTATGTGTGTGTGTGTGTGCAGGGGGTGATGTGGAATCTCCAAAGAGGGAGTCCTAATCACTGTGTGTCTTCTCATCAGAGTCTTTGTACCTGAAACTCAGGGAACATGGCTCCTCAGGTGAGGTAACCCCCGTGGGAATCACCACCTCCTCTCACCTTAA | *Orcinus orca* | 99 | 98.98 | **1.27 × 10^5^** |
| F20 | 10-May-23 | 32°09'57.5"N | 124°29'02.9"E |  |  |  |  |  |
| G01 | 11-July-23 | 32°30'00.0"N | 126°00'00.0"E |  |  |  |  |  |
| G02 | 12-July-23 | 32°30'00.0"N | 127°00'00.0"E |  |  |  |  |  |
| G03 | 12-July-23 | 32°00'00.0"N | 127°00'00.0"E |  |  |  |  |  |
| G04 | 12-July-23 | 32°00'00.0"N | 126°00'00.0"E |  |  |  |  |  |
| G05 | 12-July-23 | 31°30'00.0"N | 126°00'00.0"E |  |  |  |  |  |
| G06 | 12-July-23 | 31°30'00.0"N | 125°00'00.0"E |  |  |  |  |  |
